# Alcohol consumption and risks of more than 200 diseases in Chinese men

**DOI:** 10.1038/s41591-023-02383-8

**Published:** 2023-06-08

**Authors:** Pek Kei Im, Neil Wright, Ling Yang, Ka Hung Chan, Yiping Chen, Yu Guo, Huaidong Du, Xiaoming Yang, Daniel Avery, Shaojie Wang, Canqing Yu, Jun Lv, Robert Clarke, Junshi Chen, Rory Collins, Robin G. Walters, Richard Peto, Liming Li, Zhengming Chen, Iona Y. Millwood, China Kadoorie Biobank Collaborative Group, Chen Wang, Maxim Barnard, Derrick Bennett, Ruth Boxall, Johnathan Clarke, Ahmed Edris Mohamed, Hannah Fry, Simon Gilbert, Andri Iona, Maria Kakkoura, Christiana Kartsonaki, Hubert Lam, Kuang Lin, James Liu, Mohsen Mazidi, Sam Morris, Qunhua Nie, Alfred Pozarickij, Paul Ryder, Saredo Said, Dan Schmidt, Becky Stevens, Iain Turnbull, Baihan Wang, Lin Wang, Pang Yao, Xiao Han, Can Hou, Qingmei Xia, Chao Liu, Pei Pei, Dianjianyi Sun, Naying Chen, Duo Liu, Zhenzhu Tang, Ningyu Chen, Qilian Jiang, Jian Lan, Mingqiang Li, Yun Liu, Fanwen Meng, Jinhuai Meng, Rong Pan, Yulu Qin, Ping Wang, Sisi Wang, Liuping Wei, Liyuan Zhou, Caixia Dong, Pengfei Ge, Xiaolan Ren, Zhongxiao Li, Enke Mao, Tao Wang, Hui Zhang, Xi Zhang, Jinyan Chen, Ximin Hu, Xiaohuan Wang, Zhendong Guo, Huimei Li, Yilei Li, Min Weng, Shukuan Wu, Shichun Yan, Mingyuan Zou, Xue Zhou, Ziyan Guo, Quan Kang, Yanjie Li, Bo Yu, Qinai Xu, Liang Chang, Lei Fan, Shixian Feng, Ding Zhang, Gang Zhou, Yulian Gao, Tianyou He, Pan He, Chen Hu, Huarong Sun, Xukui Zhang, Biyun Chen, Zhongxi Fu, Yuelong Huang, Huilin Liu, Qiaohua Xu, Li Yin, Huajun Long, Xin Xu, Hao Zhang, Libo Zhang, Jian Su, Ran Tao, Ming Wu, Jie Yang, Jinyi Zhou, Yonglin Zhou, Yihe Hu, Yujie Hua, Jianrong Jin, Fang Liu, Jingchao Liu, Yan Lu, Liangcai Ma, Aiyu Tang, Jun Zhang, Liang Cheng, Ranran Du, Ruqin Gao, Feifei Li, Shanpeng Li, Yongmei Liu, Feng Ning, Zengchang Pang, Xiaohui Sun, Xiaocao Tian, Yaoming Zhai, Hua Zhang, Wei Hou, Silu Lv, Junzheng Wang, Xiaofang Chen, Xianping Wu, Ningmei Zhang, Xiaoyu Chang, Xiaofang Chen, Jianguo Li, Jiaqiu Liu, Guojin Luo, Qiang Sun, Xunfu Zhong, Weiwei Gong, Ruying Hu, Hao Wang, Meng Wang, Min Yu, Lingli Chen, Qijun Gu, Dongxia Pan, Chunmei Wang, Kaixu Xie, Xiaoyi Zhang

**Affiliations:** 1 Clinical Trial Service Unit and Epidemiological Studies Unit (CTSU), Nuffield Department of Population Health, University of Oxford, Oxford, UK; 2 Medical Research Council Population Health Research Unit (MRC PHRU), Nuffield Department of Population Health, University of Oxford, Oxford, UK; 3 Oxford British Heart Foundation Centre of Research Excellence, University of Oxford, Oxford, UK; 4 Fuwai Hospital, Chinese Academy of Medical Sciences, Beijing, China; 5 NCD Prevention and Control Department, Qingdao CDC, Qingdao, China; 6 Department of Epidemiology and Biostatistics, School of Public Health, Peking University, Beijing, China; 7 Peking University Center for Public Health and Epidemic Preparedness & Response, Beijing, China; 8 China National Center for Food Safety Risk Assessment, Beijing, China; 9 WHO Collaborating Center for Tobacco Cessation and Respiratory Diseases Prevention, China-Japan Friendship Hospital, Beijing, China; 10 Institute of Respiratory Medicine, Chinese Academy of Medical Sciences, Beijing, China; 11 NCD Prevention and Control Department, Guangxi Provincial CDC, Nanning, China; 12 NCD Prevention and Control Department, Liuzhou CDC, Liubei, Liuzhou, China; 13 NCD Prevention and Control Department, Gansu Provincial CDC, Lanzhou, China; 14 NCD Prevention and Control Department, Maijixiang CDC, Maijixiang, Tianshui, China; 15 NCD Prevention and Control Department, Hainan Provincial CDC, Haikou, China; 16 NCD Prevention and Control Department, Meilan CDC, Meilan, Haikou, China; 17 NCD Prevention and Control Department, Heilongjiang CDC, Harbin, China; 18 NCD Prevention and Control Department, Nangang CDC, Harbin, China; 19 NCD Prevention and Control Department, Henan Provincial CDC, Zhengzhou, China; 20 NCD Prevention and Control Department, Huixian CDC, Huixian, China; 21 NCD Prevention and Control Department, Hunan Provincial CDC, Changsha, China; 22 NCD Prevention and Control Department, Liuyang CDC, Liuyang, China; 23 NCD Prevention and Control Department, Jiangsu Provincial CDC, Nanjing, China; 24 NCD Prevention and Control Department, Wuzhong CDC, Wuzhong, Suzhou, China; 25 NCD Prevention and Control Department, Licang CDC, Qingdao, China; 26 NCD Prevention and Control Department, Sichuan Provincial CDC, Chengdu, China; 27 NCD Prevention and Control Department, Pengzhou CDC, Pengzhou, Chengdu, China; 28 NCD Prevention and Control Department, Zhejiang Provincial CDC, Hangzhou, China; 29 NCD Prevention and Control Department, Tongxiang CDC, Tongxiang, China

**Keywords:** Risk factors, Epidemiology, Genetics research

## Abstract

Alcohol consumption accounts for ~3 million annual deaths worldwide, but uncertainty persists about its relationships with many diseases. We investigated the associations of alcohol consumption with 207 diseases in the 12-year China Kadoorie Biobank of >512,000 adults (41% men), including 168,050 genotyped for *ALDH2*-rs671 and *ADH1B*-rs1229984, with >1.1 million ICD-10 coded hospitalized events. At baseline, 33% of men drank alcohol regularly. Among men, alcohol intake was positively associated with 61 diseases, including 33 not defined by the World Health Organization as alcohol-related, such as cataract (*n* = 2,028; hazard ratio 1.21; 95% confidence interval 1.09–1.33, per 280 g per week) and gout (*n* = 402; 1.57, 1.33–1.86). Genotype-predicted mean alcohol intake was positively associated with established (*n* = 28,564; 1.14, 1.09–1.20) and new alcohol-associated (*n* = 16,138; 1.06, 1.01–1.12) diseases, and with specific diseases such as liver cirrhosis (*n* = 499; 2.30, 1.58–3.35), stroke (*n* = 12,176; 1.38, 1.27–1.49) and gout (*n* = 338; 2.33, 1.49–3.62), but not ischemic heart disease (*n* = 8,408; 1.04, 0.94–1.14). Among women, 2% drank alcohol resulting in low power to assess associations of self-reported alcohol intake with disease risks, but genetic findings in women suggested the excess male risks were not due to pleiotropic genotypic effects. Among Chinese men, alcohol consumption increased multiple disease risks, highlighting the need to strengthen preventive measures to reduce alcohol intake.

## Abstract (web summary)

Observational analyses from the China Kadoorie Biobank found that alcohol consumption was associated with higher risks of 61 diseases in Chinese men, with most of these associations confirmed by genetic analyses.

## Main

Alcohol consumption is a major risk factor for poor physical and mental health, accounting for about 3 million deaths and over 130 million disability-adjusted life years worldwide in 2016 (ref. ^1^). Since the 1990s, alcohol consumption has increased in many low- and middle-income countries, including China, where it almost exclusively involves men^2,3^. Among Chinese men, those who reported alcohol consumption in the past 12 months increased from 59% to 85% and yearly per-capita alcohol consumption increased from 7.1 to 11.2 l between 1990 and 2017 and these have been predicted to increase in future years^2^.

Previous epidemiological studies conducted in mainly western populations have provided consistent evidence about the hazards of alcohol drinking for several major diseases, including several types of cancers and cardiovascular diseases (CVDs), liver cirrhosis, infectious diseases (for example tuberculosis and pneumonia) and injuries^4–9^. Large western cohort studies with linkage to hospital records have also investigated the associations of alcohol with risks of several less-common or non-fatal disease outcomes (for example certain site-specific cancers^10–12^, dementia^13^, falls^14^ and cataract surgery^15^). For some (for example stomach cancer), there was suggestive evidence of weak positive associations with heavy drinking^10,11^, whereas for others (for example cataract) the limited available evidence has been contradictory^10,12,13,15^; however, the evidence from western populations, even for diseases known to be associated with alcohol, may not be generalizable to Chinese populations, where the prevalence and types of alcohol drinking (mainly spirits), patterns of diseases (for example high stroke rates) and differences in the ability to metabolize alcohol^8,9,16^ differ markedly from those in western populations^4,17^.

For many diseases, including those considered by the World Health Organization (WHO)^4^ to be alcohol-related (for example ischemic heart disease (IHD) and diabetes), uncertainty remains about the causal relevance of these associations, which can be assessed in genetic studies using a Mendelian randomization (MR) approach^18^. In such studies, genetic variants can be used as instruments for alcohol consumption to investigate the potential causal relevance of alcohol drinking for diseases, which can limit the biases of confounding and reverse causality common in conventional observational studies^18^. Such studies are particularly informative in East Asian populations where two common genetic variants (*ALDH2*-rs671 and *ADH1B*-rs1229984), which are both rare in western populations, greatly alter alcohol metabolism and strongly affect alcohol intake^19^. Several studies have explored the causal relevance of alcohol consumption with CVD risk factors and morbidity^19–22^ and cancer^16^ using these genetic variants, yet findings remain inconclusive for certain diseases (for example IHD) and evidence for other diseases is sparse.

To address these questions, we conducted analyses using observational and genetic approaches to evaluate the associations between alcohol consumption and the risks of a wide range of disease outcomes in the prospective China Kadoorie Biobank (CKB).

## Results

Among the 512,724 participants (Supplementary Fig. 1), the mean age at baseline was 52 (s.d. 10.7) years, 41% were men and 56% lived in rural areas. Among men, 33% reported drinking alcohol regularly (at least once a week) at baseline (current drinkers), consuming on average 286 g of alcohol per week, mainly from spirits (Supplementary Tables 1 and 2). Non-drinkers and ex-drinkers were older and more likely to report poor self-rated health or previous chronic diseases, compared to occasional or current drinkers (Table 1). Compared to moderate drinkers (<140 g per week), heavier drinkers were more likely to be rural residents, had received lower education and had more unhealthy lifestyle factors (for example smoking and infrequent fresh fruit intake), higher mean blood pressure and longer duration of drinking (Supplementary Table 3). Among male current drinkers, 62% reported drinking daily and 37% engaging in heavy episodic drinking (Supplementary Table 2). Among women, only 2% drank alcohol at least weekly (mean intake 116 g per week), but there were similar associations with other baseline characteristics (Table 1 and Supplementary Tables 3 and 4) compared to those in men.

**Table 1:** Baseline characteristics by alcohol consumption status, in men and women

					Current drinkers				
	Overall	Non-drinkers	Ex-drinkers	Occasional drinkers	All current drinkers	<140 (men)/<70 (women) g per week	140–279 (men) /70–139 (women) g per week	280–419 (men) /≥140 (women) g per week	≥420 (men) g per week
Men, *n* (%)	210,205	42,779 (20%)	18,295 (9%)	79,231 (38%)	69,900 (33%)	25,093 (12%)	18,907 (9%)	12,832 (6%)	13,068 (6%)
Sociodemographic characteristics									
Mean age, years (s.d.)	52.8 (10.9)	57.0 (11.1)	56.8 (10.3)	51.0 (10.8)	51.5 (10.2)	51.3 (10.9)	51.9 (10.2)	51 (9.6)	50.7 (9.5)
Urban, %	43.5	31.2	41.1	44.1	50.0	58.6	52.7	48.5	31.2
Education >6 years, %	57.8	54.5	56.7	60.5	57.6	63.9	60.1	59.6	55.7
Household income > 20,000 yuan per year, %	45.6	42.0	44.9	46.7	46.8	53.1	51.3	49.6	50.4
Lifestyle risk factors									
Current smokers, %	61.1	52.3	60.4	56.9	71.7	64.6	72.1	76.1	79.6
Infrequent fresh fruit intake, %	77.0	75.1	74.7	74.8	78.9	72.0	78.0	81.1	83.6
Physical activity, mean MET-h/d (s.d.)	22.0 (15.3)	21.1 (15.1)	20.3 (14.5)	22.5 (15.6)	22.2 (15)	22.6 (14.5)	23.1 (14.9)	23.1 (15.4)	22.5 (15.2)
Mean systolic blood pressure, mm Hg (s.d.)	132.8 (20)	132 (21.5)	134.1 (21.5)	131 (18.8)	134.3 (19.8)	131.8 (18.9)	134.3 (19.8)	136 (20)	137.7 (20.7)
Mean body mass index, kg m^–2^ (s.d.)	23.4 (3.2)	23.3 (3.2)	23.9 (3.4)	23.4 (3.2)	23.4 (3.2)	23.7 (3.2)	23.7 (3.2)	23.7 (3.2)	23.8 (3.2)
Self-reported medical history, %									
Poor self-reported health	8.9	12.8	17.1	7.7	5.9	6.5	6.4	6.1	7.1
Previous chronic disease^a^	22.6	27.3	37.8	21.2	17.9	19.8	18.0	17.3	17.5
Women, *n* (%)	302,519	192,333 (64%)	2,657 (1%)	101,285 (33%)	6,244 (2%)	3,224 (1%)	1,587 (0.5%)	1,433 (0.5%)	
Sociodemographic characteristics									
Mean age, years (s.d.)	51.5 (10.5)	52.7 (10.7)	55.2 (9.4)	49.3 (9.9)	52.9 (10.3)	53 (10.7)	53.3 (10.1)	51.6 (9.5)	
Urban, %	44.6	42.7	30.2	48.1	46.5	60.9	34.4	23.5	
Education >6 years, %	43.3	41.2	46.5	49.0	48.2	49.6	45.4	43.5	
Household income > 20,000 yuan per year, %	40.7	38.0	44.8	44.2	47.0	41.1	36.7	36.6	
Lifestyle risk factors									
Current smokers, %	2.4	1.9	5.4	2.8	7.9	10.0	15.6	25.8	
Infrequent fresh fruit intake, %	68.2	70.0	56.4	63.1	60.9	55.8	63.0	66.1	
Physical activity, mean MET-h/d (s.d.)	20.4 (12.8)	20.1 (13.3)	20.2 (11.1)	20.6 (11.7)	20.5 (11.6)	20 (11.5)	19.7 (11.8)	19 (11.5)	
Mean systolic blood pressure, mm Hg (s.d.)	129.9 (22)	130.8 (22.5)	129.1 (23.2)	127.9 (20.5)	127.8 (21.6)	127.5 (20.9)	128.2 (21.8)	128.7 (22.5)	
Mean body mass index, kg m^–2^ (s.d.)	23.8 (3.5)	23.9 (3.5)	24 (3.5)	23.8 (3.4)	23.7 (3.4)	23.8 (3.4)	23.6 (3.3)	23.6 (3.4)	
Self-reported medical history, %									
Poor self-reported health	11.4	12.6	21.5	9.6	8.0	10.8	9.8	10.1	
Previous chronic disease^a^	22.1	23.2	32.7	20.8	19.8	23.8	22.2	21.1	

MET-h/d, metabolic equivalent of task per hour per day.

Means and percentages are adjusted for the age and study area structure of the CKB population for the four main drinking groups, and of the CKB drinker population for the weekly intake groups, using direct standardization separately by sex.

^a^Chronic diseases included self-reported history of coronary heart disease, stroke, transient ischemic attack, diabetes, tuberculosis, emphysema/chronic bronchitis, liver cirrhosis/chronic hepatitis, peptic ulcer, gallstone/gallbladder disease, kidney disease, rheumatoid arthritis and cancer.

During a median of 12.1 (interquartile range 11.1–13.1) years of follow-up, 134,641 men (44,027 drinkers) and 198,430 women (4,420 drinkers) experienced at least one reported hospitalization event or death at age-at-risk 35–84 years, involving a total of 1,111,495 hospitalization episodes. Among men, there were 333,541 (107,857 in current drinkers) recorded events from 207 diseases across 17 International Classification of Diseases Tenth Revision (ICD-10) chapters studied that had at least 80 cases each among current drinkers (Table 2), while among women there were 476,986 (11,773) events from 48 diseases across 18 ICD-10 chapters (Supplementary Table 5).

**Table 2:** Summary of number of diseases associated with alcohol consumption by ICD-10 chapter, in men

				Ever-regular versus occasional drinking		Dose–response associations among current drinkers		Total^a^	
ICD-10 chapter	No. of diseases	Total no. of events	No. of events in current drinkers	Positive	Negative	Positive	Negative	Positive	Negative
I Infectious and parasitic	7	8,010	2,501	1	0	1	0	1	0
II Neoplasms	27	26,064	9,437	9	0	5	0	10	0
III Blood and immune-related	3	1,596	527	0	0	2	0	2	0
IV Endocrine, nutritional and metabolic	6	15,645	5,227	1	1	2	0	2	1
V Psychiatric and behavioral	3	1,683	587	0	0	1	0	1	0
VI Nerve-related	7	7,649	2,574	2	0	1	0	2	0
VII Eye and adnexa	7	10,142	3,240	0	0	1	0	1	0
VIII Ear and mastoid process	3	2,131	667	0	0	0	0	0	0
IX Circulatory	26	101,913	30,784	9	0	10	0	14	0
X Respiratory	23	42,993	13,233	3	0	1	0	3	0
XI Digestive	29	39,118	13,624	6	0	6	1	9	1
XII Skin and subcutaneous tissue	4	2,241	789	0	0	1	0	1	0
XIII Musculoskeletal	15	21,240	6,892	3	0	2	0	3	0
XIV Genitourinary	15	18,422	5,419	0	0	0	1	0	1
XVIII Other symptoms, signs and abnormal findings	16	19,881	7,191	2	0	4	0	4	0
XIX Injury, poisoning and other external causes	12	12,509	4,402	3	0	1	0	4	0
XX External causes	4	2,304	763	3	0	4	0	4	0
Total	207	333,541	107,857	42^b^	1	42^c^	2	61^d^	3

^a^Included disease associations from Cox regression analyses either significant (*P* < 0.05 for diseases classified as alcohol-related by the WHO, FDR-adjusted *P* < 0.05 for other diseases; two-sided) from the comparison of ever-regular versus occasional drinking or dose–response association analyses within current drinkers.

^b^Of the 42 diseases showing significant positive associations with ever-regular versus occasional drinking, 23 diseases were classified as alcohol-related by the WHO.

^c^Of the 42 diseases showing significant positive dose–response associations among current drinkers, 22 diseases were classified as alcohol-related by the WHO.

^d^Of the 61 diseases showing significant positive associations with alcohol, 28 diseases were classified as alcohol-related by the WHO (of which 25 diseases passed FDR significance at FDR-adjusted *P* < 0.05 and 3 diseases were nominally significant at *P* < 0.05).

### Observational associations of alcohol with disease risks

**Fig. 1:**
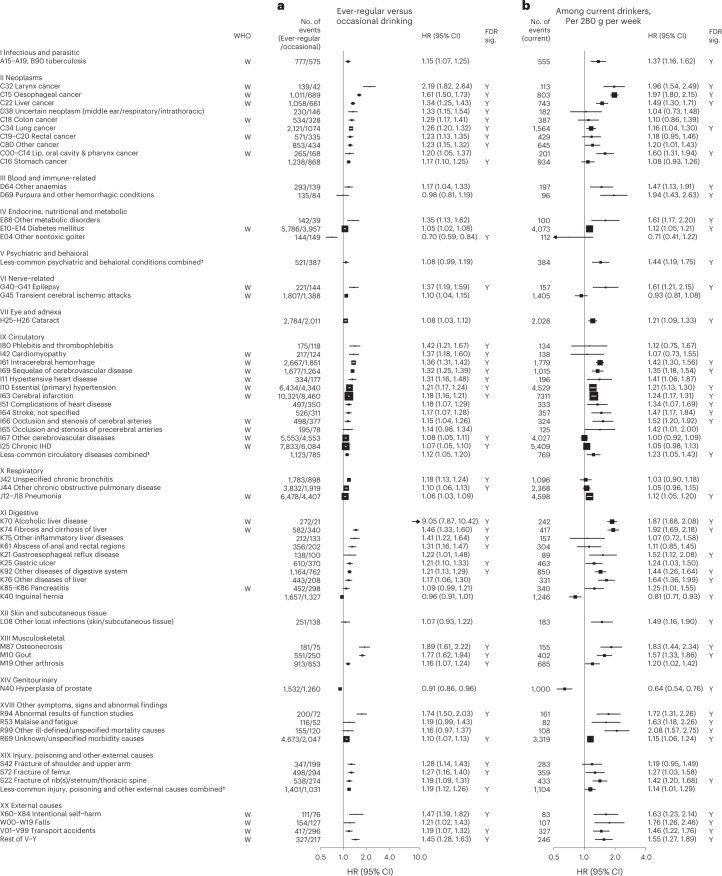
Adjusted HRs for specific diseases showing significant associations with alcohol consumption by ICD-10 chapters, in men. Cox models (**a**) comparing ever-regular drinkers with occasional drinkers or (**b**) assessing the dose–response per 280 g per week higher usual alcohol intake within current drinkers, were stratified by age at risk and study area and were adjusted for education and smoking. Each solid square represents HR with the area inversely proportional to the variance of the log HR. The horizontal lines indicate 95% CIs. Diseases considered to be alcohol-related by the WHO are indicated with ‘W’ under the ‘WHO’ column. The individual diseases listed included all that showed FDR-adjusted significant associations with alcohol (FDR-adjusted *P* < 0.05, indicated with ‘Y’ under the ‘FDR sig.’ column) and WHO alcohol-related diseases that showed nominally significant associations with alcohol (*P* < 0.05). All *P* values are two-sided. † Included less-common ICD-10 codes within the corresponding ICD-10 chapter that were not individually investigated in the present study. ‘Less-common psychiatric and behavioral conditions’ consisted of ICD-10 codes F00–F99, excluding F32, F33 and F99. ‘Less-common circulatory diseases’ consisted of ICD-10 codes I00–I99, excluding I10, I11, I20, I21, I24, I25, I27, I42, I46, I48–I51, I60–I67, I69, I70, I80 and I83. ‘Less-common injury, poisoning and other external causes’ consisted of ICD-10 codes S00–T98, excluding S06, S09, S22, S32, S42, S52, S62, S72, S82, S92 and T14.

**Extended Data Fig. 1:**
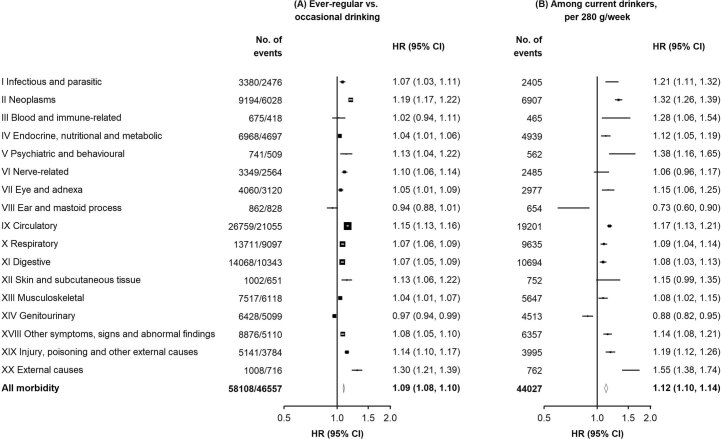
Adjusted HRs for ICD−10 chapter−specific morbidities associated with ever-regular drinking and with usual alcohol intake, in men. Cox models comparing ever-regular drinkers with occasional drinkers, or assessing the dose–response per 280 g/week higher usual alcohol intake within current drinkers, were stratified by age-at-risk and study area and adjusted for education and smoking. Each solid square or diamond represents HR with the area inversely proportional to the variance of the log HR. The horizontal lines indicate 95% CIs. CI, confidence interval; HR hazard ratio; ICD-10, International Classification of Diseases, 10th Revision.

**Extended Data Fig. 2:**
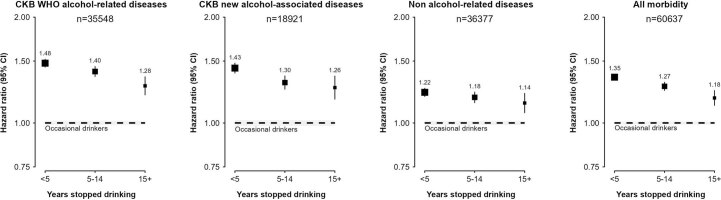
Adjusted HRs for different aggregated and all-cause morbidities associated with years after stopping drinking, in men. Cox models comparing ex-drinker groups with occasional drinkers were stratified by age-at-risk and study area and were adjusted for education and smoking. Each box represents HR with the area inversely proportional to the variance of the group-specific log hazard within subplot. The vertical lines indicate group-specific 95% CIs for various ex-drinker groups. The shaded strip indicate the group-specific 95% CIs for occasional drinkers. The numbers above the error bars are point estimates for HRs. CI, confidence interval; HR hazard ratio; CKB, China Kadoorie Biobank; WHO, World Health Organization.

Among men, alcohol drinking was significantly associated with higher risks of 61 disease outcomes from 15 ICD-10 chapters based on two separate analyses, (1) comparing ever-regular versus occasional drinkers and (2) dose–response among current drinkers (Table 2 and Extended Data Fig. 1). In each of the analyses in men, there were significant associations of alcohol consumption with 42 diseases (or outcomes), of which 23 were significant in both analyses and the remainder were directionally consistent with one exception (transient cerebral ischemic attacks, ICD-10 code G45) (Fig. 1). In further analyses covering all alcohol consumption categories, there were typical U-shaped or J-shaped associations, with excess risks in male ex-drinkers and non-drinkers compared to occasional or moderate drinkers for most of these diseases (Supplementary Table 6). Among male ex-drinkers, the overall excess morbidity risks were more considerable for alcohol-associated diseases than for other diseases, but these excess risks were lower with increasing duration after stopping drinking (Extended Data Fig. 2).

**Extended Data Fig. 3:**
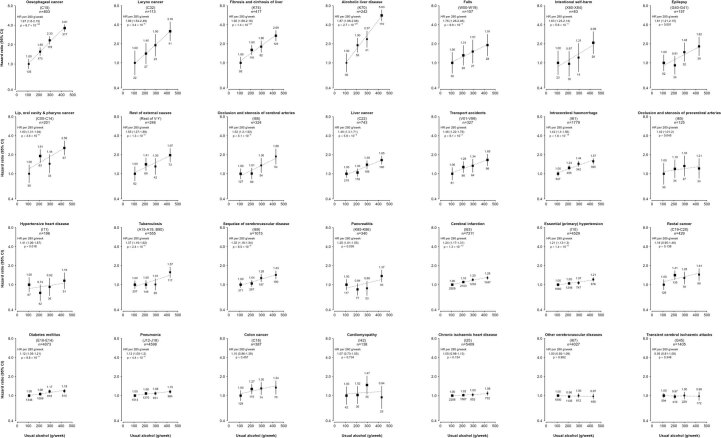
Associations of alcohol consumption with risks of 28 diseases previously defined as alcohol-related by the WHO, in male current drinkers. Cox models were stratified by age-at-risk and study area and were adjusted for education and smoking. HRs were plotted against usual alcohol intake and were calculated per 280 g/week higher usual alcohol intake. All specific diseases displayed were significantly associated with alcohol intake (ever-regular drinking or per 280 g/week higher usual alcohol intake) after multiple testing correction (FDR-adjusted p<0.05), except transient cerebral ischemic attacks and related syndromes (ICD-10 code: G45), occlusion and stenosis of precerebral arteries (I65) and pancreatitis (K85-K86) which showed statistical significance at nominal level (p<0.05). Each box represents HR with the area inversely proportional to the variance of the group-specific log hazard within subplot. The vertical lines indicate group-specific 95% CIs. The numbers above the error bars are point estimates for HRs and the numbers below are number of events. All P values are two-sided. CI, confidence interval; HR hazard ratio; CKB, China Kadoorie Biobank; FDR, false discovery rate; ICD-10, International Classification of Diseases, 10th Revision; WHO, World Health Organization.

Of the 61 diseases positively associated with alcohol intake in male participants, 28 were considered by the WHO to be alcohol-related diseases, including tuberculosis (A15–A19 and B90), six site-specific cancers including cancers in the larynx (C32), esophagus (C15), liver (C22), colon (C18), rectum (C19 and C20) and lips, oral cavity and pharynx (C00–C14), diabetes (E10–E14), epilepsy (G40 and G41), several hypertensive diseases (I10 and I11) and cerebrovascular diseases (I61, I63, I65, I66, I67, I69 and G45), chronic IHD (I25), cardiomyopathy (I42), pneumonia (J12–J18), alcoholic liver disease (K70) and liver cirrhosis (K74), pancreatitis (K85 and K86) and external causes including self-harm (X60–X84), falls (W00–W19), transport accidents (V01–V99) and other external causes (rest of V–Y) (Fig. 1 and Extended Data Fig. 3). Of these 28 diseases, 22 showed significant dose–response associations with alcohol intake. The hazard ratios (HRs) per 280 g per week higher intake for the aggregated WHO alcohol-related diseases were 1.22 (95% confidence interval (CI) 1.19–1.25) (Supplementary Table 7 for detailed outcome classification), ranging from 1.12 (1.05–1.20) for pneumonia to 1.97 (1.80–2.15) for esophageal cancer.

**Extended Data Fig. 4:**
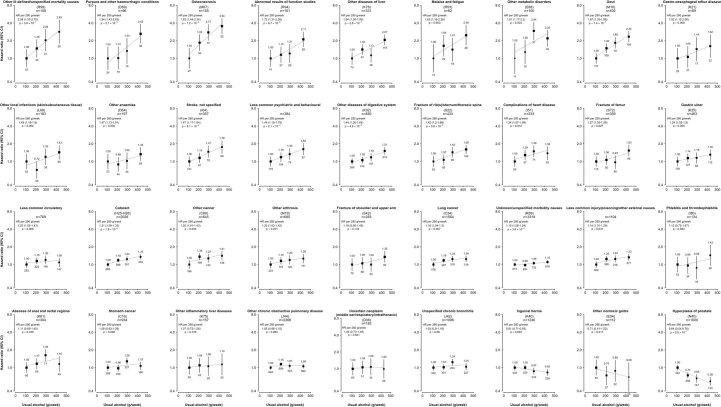
Associations of alcohol consumption with risks of 36 diseases not previously defined as alcohol-related, in male current drinkers. Cox models were stratified by age-at-risk and study area and were adjusted for education and smoking. HRs were plotted against usual alcohol intake and were calculated per 280 g/week higher usual alcohol intake. All specific diseases displayed were significantly associated with alcohol intake (ever-regular drinking or per 280 g/week higher usual alcohol intake) after multiple testing correction (FDR-adjusted p<0.05). Each box represents HR with the area inversely proportional to the variance of the group-specific log hazard within subplot. The vertical lines indicate group-specific 95% CIs. The numbers above the error bars are point estimates for HRs and the numbers below are number of events. All P values are two-sided. CI, confidence interval; HR hazard ratio; CKB, China Kadoorie Biobank; FDR, false discovery rate.

The 33 other diseases showing false discovery rate (FDR)-adjusted significant positive associations with alcohol drinking in men included lung (C34) and stomach (C16) cancers, cataract (H25 and H26), six digestive diseases such as gastroesophageal reflux disease (K21) and gastric ulcer (K25), three musculoskeletal conditions, including gout (M10), three fracture types (S22, S42 and S72), and the aggregates of less-common psychiatric and behavioral conditions and circulatory diseases (Fig. 1 and Extended Data Fig. 4). Of these 33 diseases, 22 showed significant dose–response associations, with HRs per 280 g per week higher intake ranging from 1.16 (95% CI 1.04–1.30) for lung cancer to 1.94 (1.43–2.63) for purpura and other hemorrhagic conditions (D69) and 1.20 (1.16–1.24) for the aggregated CKB new alcohol-associated diseases. In contrast, three diseases showed FDR-adjusted significant inverse associations with alcohol drinking (other nontoxic goiter (E04), hyperplasia of prostate (N40) and inguinal hernia (K40)). Overall, for all-cause morbidity, the HR per 280 g per week higher intake was 1.12 (1.10–1.14) in male current drinkers.

Supplementary Figs. 2–4 show the dose–response associations for all disease outcomes investigated in male current drinkers. For alcohol-associated diseases and for total morbidity, the dose–response associations were unaltered after additional covariate adjustments or excluding participants with poor baseline health conditions (Supplementary Fig. 5 and Supplementary Table 8). Moreover, the associations were similar across various male population subgroups, but seemed to be stronger in younger men, urban residents and higher socioeconomic groups for new alcohol-associated diseases (Supplementary Fig. 6).

**Extended Data Fig. 5:**
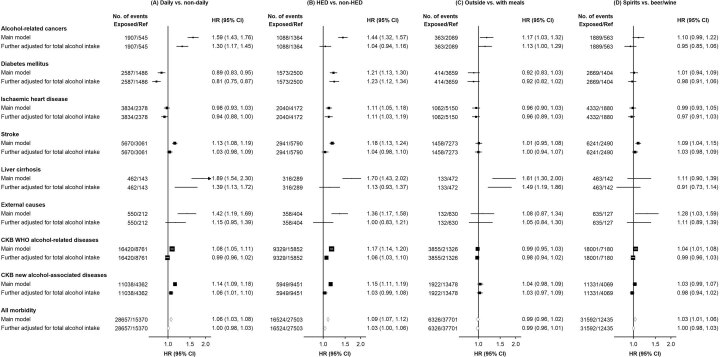
Adjusted HRs for major diseases associated with drinking patterns, in male current drinkers. Cox models were stratified by age-at-risk and study area and were adjusted for education and smoking and for total alcohol intake where indicated. Each solid square or diamond represents HR with the area inversely proportional to the variance of the log HR. The horizontal lines indicate 95% CIs. CI, confidence interval; HR hazard ratio; HED, heavy episodic drinking; CKB, China Kadoorie Biobank; WHO, World Health Organization.

**Extended Data Fig. 6:**
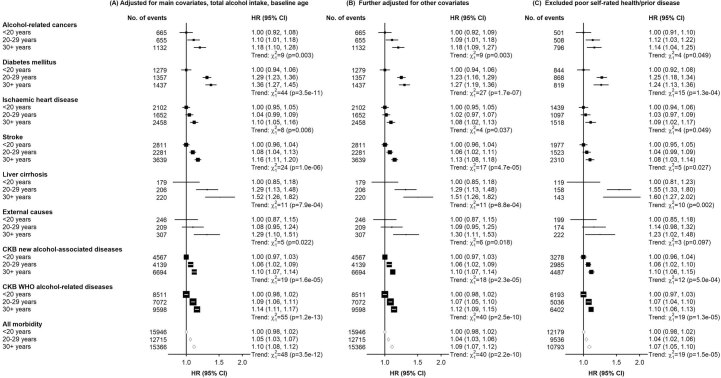
Adjusted HRs for major diseases associated with duration of drinking, in male current drinkers. Cox models were stratified by age-at-risk and study area and were adjusted for education, smoking, total alcohol intake and baseline age in (A). (B) had the same model specifications as (A) plus further adjustments for income, physical activity, fruit intake and body mass index. (C) had the same model specifications as (A) and excluded participants with poor self-reported health or prior chronic disease at baseline. Each box represents HR with the area inversely proportional to the variance of the group-specific log hazard. The horizontal lines indicate group-specific 95% CIs. All P values are two-sided. CI, confidence interval; HR hazard ratio; CKB, China Kadoorie Biobank; WHO, World Health Organization.

Among male current drinkers, drinking daily, heavy episodic drinking and drinking spirits were each associated with higher risks for alcohol-related diseases, but most of these associations were attenuated to the null after adjusting for total alcohol intake (Extended Data Fig. 5); however, for a given total alcohol intake among male current drinkers, drinking daily was associated with 30–40% higher risks of alcohol-related cancers (1.30, 1.17–1.45) and liver cirrhosis (1.39, 1.13–1.72), compared to non-daily drinking. Similarly, heavy episodic drinking was associated with higher risks of diabetes (1.23, 1.12–1.34) and IHD (1.11, 1.03–1.19), whereas drinking outside of meals was associated with 49% (1.49, 1.19–1.86) higher risk of liver cirrhosis than drinking with meals. The risks of all major alcohol-associated diseases were higher with longer duration of alcohol consumption in men (Extended Data Fig. 6).

**Extended Data Fig. 7:**
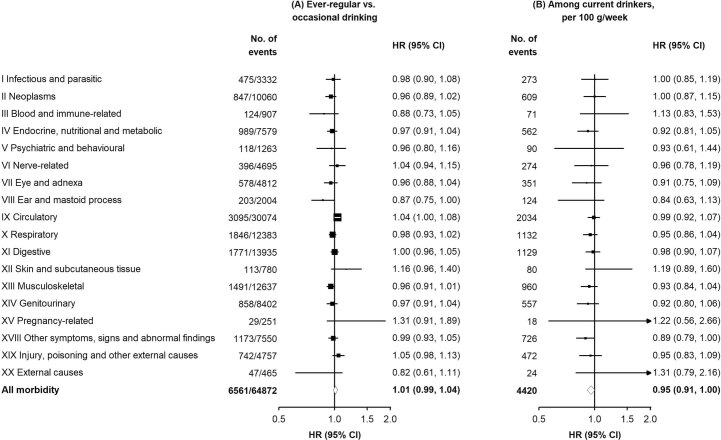
Adjusted HRs for ICD−10 chapter−specific morbidities associated with ever-regular drinking and with usual alcohol intake, in women. Cox models comparing ever-regular drinkers with occasional drinkers, or assessing the dose–response per 100 g/week higher usual alcohol intake within current drinkers, were stratified by age-at-risk and study area and adjusted for education and smoking. Each solid square or diamond represents HR with the area inversely proportional to the variance of the log HR. The horizontal lines indicate 95% CIs. CI, confidence interval; HR hazard ratio; ICD-10, International Classification of Diseases, 10th Revision.

Among women, due to few reported current drinkers there was a lack of statistical power to detect any associations of self-reported alcohol intake with disease risks (Supplementary Table 5, Extended Data Fig. 7 and Supplementary Fig. 7).

### Genetic associations of alcohol with disease risks

A genetic instrument for alcohol intake was derived using *ALDH2*-rs671 (G > A) and *ADH1B*-rs1229984 (G > A) genotypes. The overall A-allele frequency was 0.21 for *ALDH2*-rs671 and 0.69 for *ADH1B*-rs1229984, with both A-alleles being more common in southern than northern study areas (Supplementary Table 9). Both *ALDH2*-rs671 and, to a lesser extent, *ADH1B*-rs1229984 were strongly associated with alcohol drinking in men, but much less so in women (Supplementary Table 10). In men, the derived genetic instrument predicted a >60-fold difference (range 4–255 g per week, C1 to C6) in mean alcohol intake, whereas in women mean alcohol intake remained low (<10 g per week) across genetic categories (Supplementary Table 11). Both variants and the derived instrument were not associated with smoking or other major self-reported baseline characteristics, except for a small difference in fresh fruit intake by *ALDH2*-rs671 genotype in men.

**Fig. 2:**
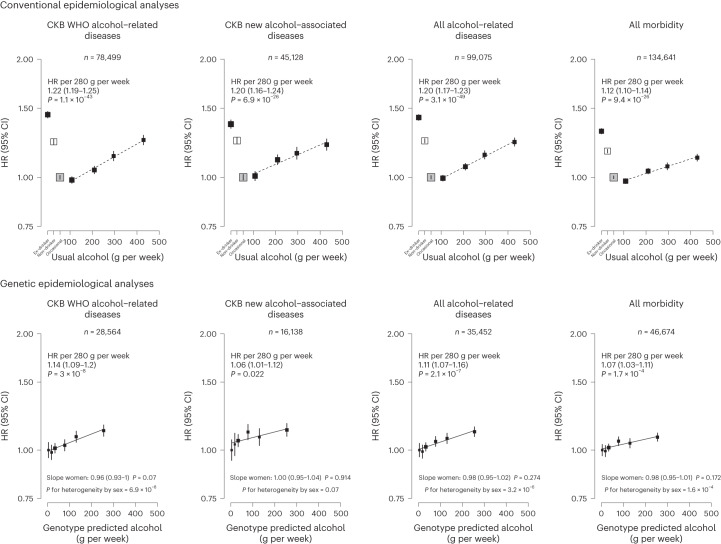
Associations of alcohol-related diseases and overall morbidity with self-reported alcohol intake and with genotype-predicted mean alcohol intake, in men. Each box represents HR with the area inversely proportional to the variance of the group-specific log hazard within subplot. The vertical lines indicate group-specific 95% CIs. Conventional epidemiological analyses relate self-reported drinking patterns to risks of diseases (reference group is occasional drinkers), using Cox models stratified by age at risk and study area and adjusted for education and smoking. Within current drinkers, HRs were plotted against usual alcohol intake and were calculated per 280 g per week higher usual alcohol intake. Genetic epidemiological analyses relate genetic categories to risks of diseases (reference group is the genotype group with lowest genotype-predicted mean male alcohol intake), using Cox models stratified by age at risk and study area and adjusted for genomic principal components. The HR per 280 g per week higher genotype-predicted mean male alcohol intake was calculated from the inverse-variance-weighted mean of the slopes of the fitted lines in each study area. The corresponding slopes in women were summarized in text and the slopes of the fitted line by sex were compared and assessed for heterogeneity using chi-squared tests (indicated by *P* for heterogeneity by sex). All *P* values are two-sided. Analyses of these aggregated outcomes were based on first recorded event of the aggregate during follow-up and participants may have had multiple events of different types of diseases. ‘All alcohol-related diseases’ includes the first recorded event from ‘CKB WHO alcohol-related diseases’ or ‘CKB new alcohol-associated diseases’ during follow-up.

**Fig. 3:**
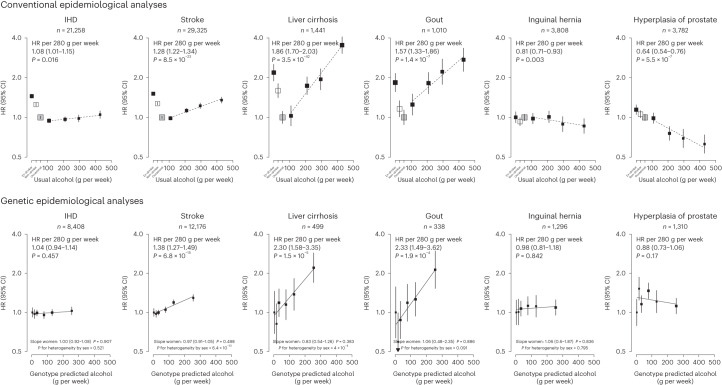
Associations of selected alcohol-related diseases with self-reported alcohol intake and with genotype-predicted mean alcohol intake, in men. Each box represents HR with the area inversely proportional to the variance of the group-specific log hazard within subplot. The vertical lines indicate group-specific 95% CIs. Conventional epidemiological analyses relate self-reported drinking patterns to risks of diseases (reference group is occasional drinkers), using Cox models stratified by age at risk and study area and adjusted for education and smoking. Within current drinkers, HRs were plotted against usual alcohol intake and were calculated per 280 g per week higher usual alcohol intake. Genetic epidemiological analyses relate genetic categories to risks of diseases (reference group is the genotype group with lowest genotype-predicted mean male alcohol intake), using Cox models stratified by age at risk and study area and adjusted for genomic principal components. The HR per 280 g per week higher genotype-predicted mean male alcohol intake was calculated from the inverse-variance-weighted mean of the slopes of the fitted lines in each study area. The corresponding slopes in women were summarized in text and the slopes of the fitted line by sex were compared and assessed for heterogeneity using chi-squared tests (indicated by *P* for heterogeneity by sex). All *P* values are two-sided. Corresponding ICD-10 codes, IHD (I20–I25); stroke (I60, I61, I63 and I64); liver cirrhosis (K70 and K74); gout (M10); inguinal hernia (K40); hyperplasia of prostate (N40).

**Extended Data Fig. 8:**
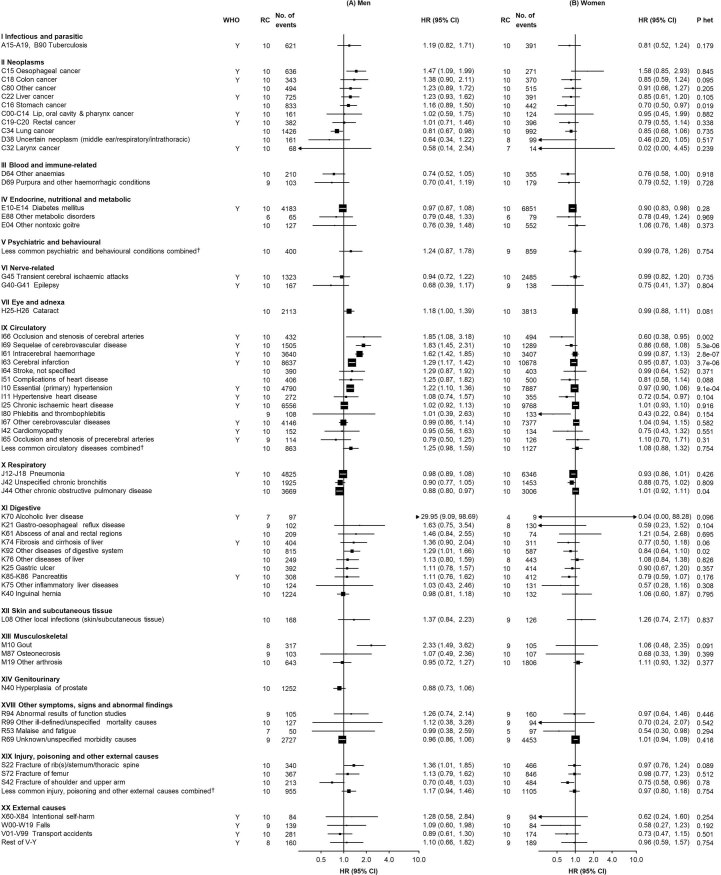
Adjusted HRs per 280 g/week higher genotype-predicted mean male alcohol intake for specific alcohol-associated diseases by ICD-10 chapters, in men and women. Cox modes, stratified by age-at-risk and adjusted for genomic principal components, were used to relate genetic categories to risks of diseases within each study area. The HR per 280 g/week higher genotype-predicted mean male alcohol intake was calculated from the inverse-variance-weighted mean of the slopes of the fitted lines in each study area. Each solid square or diamond represents HR per 280 g/week higher genetically-predicted mean male alcohol intake, with the area inversely proportional to the variance of the log HR. The horizontal lines indicate 95% CIs. Diseases considered to be alcohol-related by the WHO are indicated with ‘Y’ under the ‘WHO’ column. The ‘RC’ column indicates the number of study areas that contributed to the overall area-stratified genotypic associations, as for certain less common diseases some study areas may not have enough number of cases to contribute to the inverse-variance-weighted meta-analysis. The ‘P het’ column indicates the p-value from a $\chi$^2^ test for heterogeneity between sexes. All P values are two-sided. † Included less common ICD-10 codes within the corresponding ICD-10 chapter which were not individually investigated in the present study. CI, confidence interval; HR hazard ratio; ICD-10, International Classification of Diseases, 10th Revision; WHO, World Health Organization.

**Extended Data Fig. 9:**
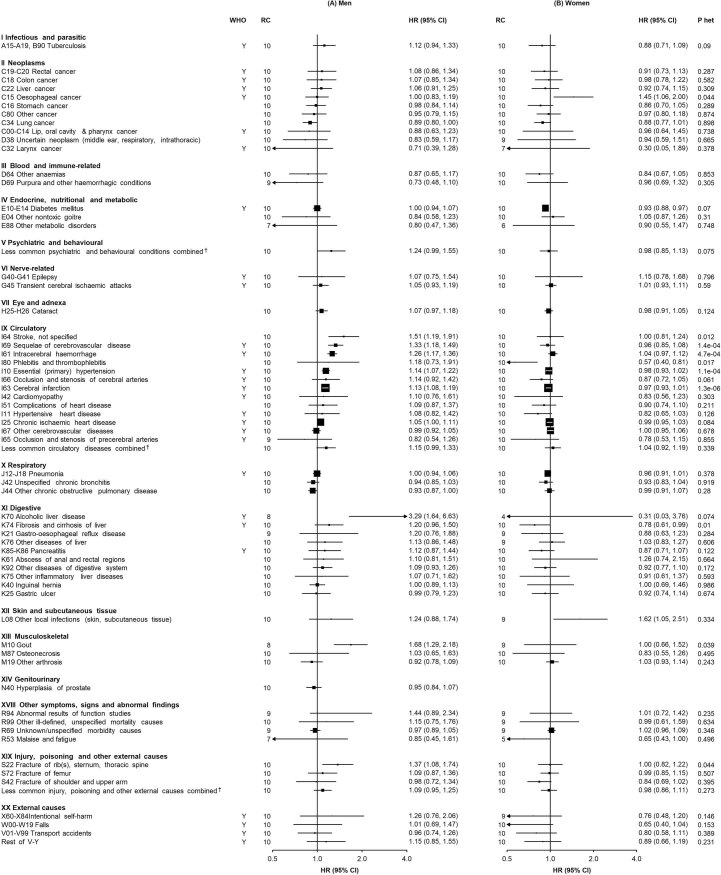
Adjusted HRs associated with GG versus AG genotype of *ALDH2*-rs671 for specific alcohol-associated diseases by ICD-10 chapters, in men and women. Area-specific genotypic effects (GG vs. AG genotype) were estimated within each study area (thus each reflecting the purely genotypic effects) using age-at-risk-stratified and genomic principal components-adjusted Cox models and were combined by inverse-variance-weighted meta-analysis to yield the overall area-stratified genotypic associations. Each solid square represents HR for GG vs. AG genotype, with the area inversely proportional to the variance of the log HR. The horizontal lines indicate 95% CIs. Diseases considered to be alcohol-related by the WHO are indicated with ‘Y’ under the ‘WHO’ column. The ‘RC’ column indicates the number of study areas that contributed to the overall area-stratified genotypic associations, as for certain less common diseases some study areas may not have enough number of cases to contribute to the inverse-variance-weighted meta-analysis. The ‘P het’ column indicates the P value from a $\chi$^2^ test for heterogeneity between sexes. All P values are two-sided. † Included less common ICD-10 codes within the corresponding ICD-10 chapter which were not individually investigated in the present study. CI, confidence interval; HR hazard ratio; ICD-10, International Classification of Diseases, 10th Revision; WHO, World Health Organization.

**Extended Data Fig. 10:**
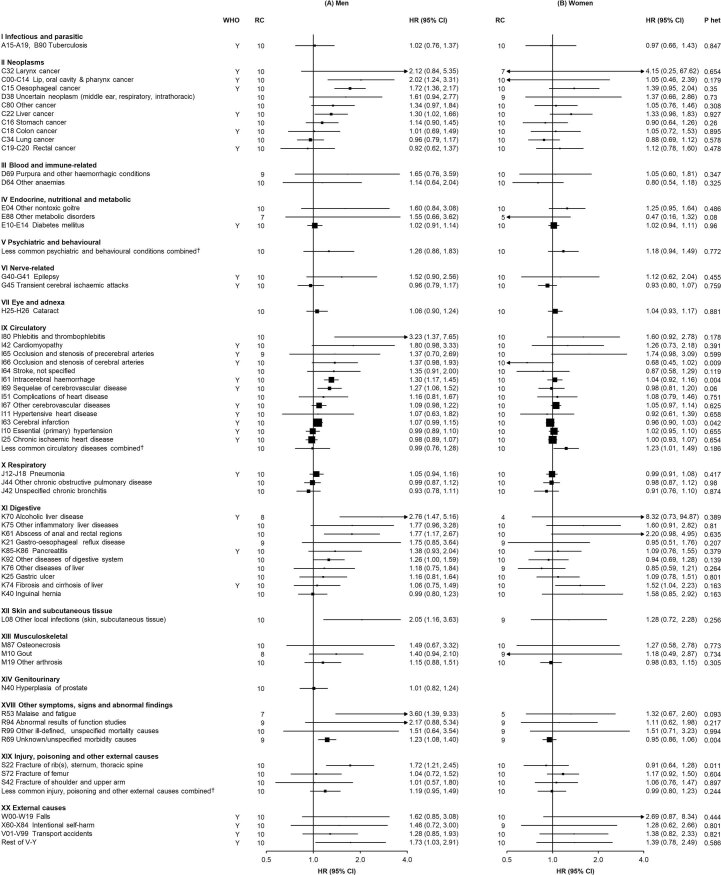
Adjusted HRs associated with GG versus AG genotype of *ADH1B*-rs1229984 for specific alcohol-associated diseases by ICD-10 chapters, in men and women. Area-specific genotypic effects (GG vs. AG genotype) were estimated within each study area (thus each reflecting the purely genotypic effects) using age-at-risk-stratified and genomic principal components-adjusted Cox models and were combined by inverse-variance-weighted meta-analysis to yield the overall area-stratified genotypic associations. Each solid square represents HR for GG vs. AG genotype, with the area inversely proportional to the variance of the log HR. The horizontal lines indicate 95% CIs. Diseases considered to be alcohol-related by the WHO are indicated with ‘Y’ under the ‘WHO’ column. The ‘RC’ column indicates the number of study areas that contributed to the overall area-stratified genotypic associations, as for certain less common diseases some study areas may not have enough number of cases to contribute to the inverse-variance-weighted meta-analysis. The ‘P het’ column indicates the P value from a $\chi$^2^ test for heterogeneity between sexes. All P values are two-sided. † Included less common ICD-10 codes within the corresponding ICD-10 chapter which were not individually investigated in the present study. CI, confidence interval; HR hazard ratio; ICD-10, International Classification of Diseases, 10th Revision; WHO, World Health Organization.

Among men, genotype-predicted mean alcohol intake was positively associated with higher risks of CKB WHO alcohol-related (HR per 280 g per week higher genotype-predicted mean male alcohol intake: 1.14, 95% CI 1.09–1.20) and CKB new alcohol-associated (1.06, 1.01–1.12) diseases (Fig. 2), both of which were slightly weaker than the conventional associations. For certain diseases, however, the genetic associations were stronger, with HRs of 1.38 (1.27–1.49) for stroke, 2.30 (1.58–3.35) for liver cirrhosis and 2.33 (1.49–3.62) for gout, in men (Fig. 3 and Extended Data Fig. 8). For individual genetic variants, the associations were directionally consistent (Extended Data Figs. 9 and 10). Conversely, there were no significant dose–response genotypic associations with IHD, inguinal hernia or hyperplasia of prostate in men. For other alcohol-associated diseases, higher genotype-predicted mean male alcohol intake was significantly associated with higher risks of esophageal cancer, cataract, occlusion and stenosis of cerebral arteries, sequelae of cerebrovascular disease, essential primary hypertension and fractures of ribs, sternum or thoracic spine. There were also suggestive positive genotypic associations with several digestive tract cancer types (liver, colon and stomach) and circulatory and digestive diseases, and significant inverse associations with lung cancer and other chronic obstructive pulmonary disease (J44) in men (Extended Data Figs. 8–10). Sensitivity analyses using different analytical methods to adjust for confounding by study area, or a two-stage least-squares MR approach, did not alter the main genetic findings in men (Supplementary Table 12). In contrast, genotypes that increased alcohol intake in men were not adversely associated with most alcohol-related disease risks among women (for example HR 1.00 (0.97–1.04) for all morbidity among female non-drinkers; Supplementary Fig. 7 and Extended Data Figs. 8–10).

### Hospitalizations associated with alcohol drinking

Among men, ever-regular drinkers had higher numbers of hospitalizations for any causes than occasional drinkers, particularly for cancer hospitalizations, and these differences increased with increasing age at risk, except for CVD hospitalizations (Supplementary Fig. 8).

## Discussion

This prospective study provides a comprehensive assessment of the impact of alcohol consumption on a very wide range of disease outcomes in Chinese adults. Among men, alcohol consumption was associated with significantly higher risks of 61 diseases, including 33 not previously reported as alcohol-related diseases by the WHO, and higher risks of hospitalizations for any causes. For a given total amount, drinking daily, heavy episodic drinking and drinking outside of meals exacerbated the risks of four major diseases in Chinese men. Moreover, most of these associations in Chinese men were confirmed in genetic analyses, at least when assessed collectively, and are likely to reflect the effects alcohol consumption itself rather than any pleiotropic effects of the genetic instruments.

Based primarily on observational findings in western populations, alcohol consumption has been considered by the WHO^4^ and the Global Burden of Disease (GBD) study^23^ to be related to about 20 distinct disease categories, involving chronic diseases and cancers largely in the gastrointestinal system, several CVD types, infectious diseases and injuries. The observational analyses largely confirmed these known associations (Supplementary Table 13), but also provided insights into additional hazards of certain drinking patterns suggested by previous studies^8,9,24,25^. Moreover, this study discovered 33 additional alcohol-associated diseases across various body systems in Chinese men that had not been previously reported by the WHO. For these 33 disease outcomes, their associations with alcohol intake were confirmed in genetic analyses, at least collectively as well as for certain specific diseases (for example gout), as was the case for a similar number of WHO alcohol-related diseases. The somewhat smaller relative (but not absolute) risks of alcohol drinking with major diseases at older than younger age in men from observational analyses were consistent with previous studies of other risk factors (for example blood pressure^26^ and smoking^27^), which could be driven by a number of factors such as selection bias^27^ and comorbidities.

For certain major WHO alcohol-related diseases, particularly IHD and ischemic stroke, observational studies, including this study, have consistently reported J-shaped associations, with those who drank moderately (for example 1–2 units a day) having the lowest risks^6,28^; however, these apparent protective effects of moderate drinking probably largely reflect residual confounding (for example non-drinkers having worse health and socioeconomic profiles than occasional drinkers) and uncontrolled reverse causation (for example sick-quitter effect where pre-existing poor health or changes in health conditions lead to alcohol cessation), including the difficulty in defining abstainers (for example ex-drinkers may be reported as non-drinkers) as the reference group in many previous studies^3,29^. In this study, we used occasional drinkers rather than non-drinkers as the reference group, which, together with separate dose–response analyses among current drinkers, helped to reduce but not eliminate any such biases, which could largely be mitigated in genetic analyses using an MR approach.

To date the existing MR studies for alcohol have focused mainly on CVD types^30–32^ and cancers^33–35^, with limited data for other diseases. Moreover, previous studies mainly involved European-ancestry populations and hence were constrained by availability of relatively weak genetic instruments. Using genetic instruments specific to East Asian populations that predicted >60-fold difference in alcohol consumption, we previously reported evidence for the causal relevance and apparent dose–response effects of alcohol consumption on upper-aerodigestive tract cancers^16^ and stroke^19^. These findings were further corroborated by subsequent European ancestry-based MR studies^30,32,36^ and the analyses presented in this study with additional follow-up data. In contrast to stroke, we found no reliable genetic evidence for a cardioprotective, nor harmful, effect of moderate drinking on risk of IHD in men, consistent with findings in other MR studies^30,32^. The present study also demonstrated a log-linear genetic association of alcohol with liver cirrhosis and suggestive positive associations for several WHO alcohol-related digestive tract cancers in men. Moreover, separate genetic analyses among women suggests that the excess risks observed among men were due chiefly to alcohol per se rather than to potential pleiotropic effects of the alcohol-related genotypes. Further larger genetic studies are required to confirm and elucidate the potential causal relevance for each of the other WHO alcohol-related diseases individually.

For the new alcohol-associated diseases identified in this study, the available prospective epidemiological evidence has been sparse and mostly confined to western populations. For gout, previous western prospective studies have reported positive associations^37,38^ and an MR study of 8,000 Korean men has also reported positive associations of alcohol consumption with hyperuricemia, a risk factor for gout^39^. The present study provides genetic evidence that alcohol drinking increases the risk of gout. Consistent with the present study, previous European-ancestry-based observational studies^40,41^ and one MR study^42^ also reported positive associations of alcohol intake with risks of several fracture types. The available prospective evidence on associations between alcohol drinking and risk of cataract has been conflicting^15,43^ and one European-ancestry-based MR study reported no genetic associations^44^. We found a significant dose–response association between alcohol and risk of cataract among Chinese men in observational analyses, which was supported by the present genetic analyses.

For several other diseases (for example gastroesophageal reflux disease and gastric ulcer), the observational findings provide additional evidence to the existing literature^5,45–47^, but the supporting genetic evidence is still constrained by limited statistical power. Similarly, our observational findings for lung and stomach cancers were generally consistent with evidence provided by previous prospective studies^7,11,48,49^; however, the causal relevance of these associations remains to be elucidated in future larger MR studies with appropriate consideration of the potential gene–environment interactions between *ALDH2*-rs671 and alcohol intake (the effect of alcohol intake on cancer risks being modified by *ALDH2*-rs671 genotype due to excessive acetaldehyde)^16^ and other aldehyde exposures^50^ in cancer risks, which might similarly affect the genetic associations for respiratory diseases and other potential acetaldehyde-related diseases. In observational analyses, we found significant inverse associations for inguinal hernia, prostate hyperplasia and other nontoxic goiter, but not for several other diseases previously inversely associated with alcohol drinking, including non-Hodgkin lymphoma^48^, kidney cancer^48^, thyroid cancer^48^ and gallstones^51^. The genetic analyses, albeit with limited power, did not provide reliable evidence supporting the inverse associations with these outcomes. Future well-powered genetic investigations are warranted for less-common diseases in different populations.

The strengths of this study include the prospective design, large sample size, detailed information on alcohol consumption and drinking patterns, completeness of follow-up and a wide range of morbidity outcomes analyzed. We were also able to assess the potential causal relevance of the associations using two powerful East Asian genetic variants. Moreover, the extremely low drinking prevalence in women (regardless of their genotypes) enabled assessment for potential pleiotropy, further supporting the genetic findings among men.

Nevertheless, the study also has limitations. First, it is still possible that heavy drinking was under-reported, which could have underestimated the hazards of heavy episodic drinking. Second, as in many population-based cohort studies, extreme problematic drinkers and certain alcohol-related disease events may be under-represented, but this should not affect the assessment of the associations of alcohol with most disease outcomes. Third, while the repeated measures of alcohol consumption available in the re-survey subsets allowed us to estimate long-term usual mean alcohol intake at the group level to account for regression dilution bias, we were unable to study the effects of longitudinal alcohol drinking trajectories on health. Fourth, we were unable or underpowered to study diseases that do not normally require hospitalization (for example dementia and depression), nor alcohol-related diseases only affecting women, given the low proportion of female drinkers (for example <70 cases of breast cancer in female drinkers). While the low female drinking prevalence in CKB was consistent with findings in a nationwide survey^52^, it is possible that women may be more likely to under-report drinking than men for cultural and social reasons. Hence our null findings in women should be interpreted with caution and not be taken as a lack of alcohol-related harms in women in general, especially in the context of rising alcohol consumption among Asian women^2^. Fifth, as spirits were the main beverage type and our genetic instrument did not distinguish between beverage types, we were unable to assess beverage-specific effects on disease risks, including wine consumption, which is uncommon in China^17^ and has been proposed as potentially cardioprotective due to other non-alcoholic components in red wine^53^. Sixth, although our genetic analyses allowed comparison of the overall genetic effects of negligible, moderate and high mean alcohol intake levels for major and overall morbidities, we had limited power to confidently clarify any small threshold effects in the low consumption end, especially for individual diseases. Finally, the genetic analyses lacked statistical power to assess the associations with several individual alcohol-associated diseases so these findings should still be viewed as hypothesis-generating.

In recent decades, several studies have estimated the alcohol-attributable disease burden, involving predominantly WHO alcohol-related diseases. These estimates were based mainly on observational evidence and included the potentially biased U- or J-shaped associations with IHD and ischemic stroke^1,23,54^. We have demonstrated in both conventional and genetic analyses that alcohol drinking is associated with hazards in a dose–response manner with a much wider range of disease outcomes than previously considered by the WHO^4^ and the GBD study^23^ and do not find any evidence for protective effects for IHD or stroke, suggesting that the actual alcohol-attributable disease burden is likely to be much greater than widely believed.

Overall, the present study demonstrated substantial hazards of alcohol consumption with a wide range of disease outcomes among Chinese men. The findings reinforce the need to lower population mean levels of alcohol consumption as a public health priority in China. Future estimation of the alcohol-attributable disease burden worldwide and in specific regions should incorporate new genetic evidence from the present and any future studies about the likely causal relevance of alcohol consumption for a broad range of disease outcomes.

## Methods

### Study population

Details of the CKB study design and methods have been previously reported^55^. Briefly, 512,724 adults aged 30–79 years were recruited from ten geographically diverse (five rural and five urban) areas across China during 2004–2008. At local study assessment clinics, trained health workers administered a laptop-based questionnaire recording sociodemographic factors, lifestyle (for example alcohol drinking, smoking, diet and physical activity) and medical history; undertook physical measurements (for example blood pressure and anthropometry); and collected a blood sample for long-term storage. Two resurveys of ~5% randomly selected surviving participants were subsequently conducted in 2008 and 2013–2014 using similar procedures.

### Ethics approval

Ethical approval was obtained from the Ethical Review Committee of the Chinese Centre for Disease Control and Prevention (Beijing, China, 005/2004) and the Oxford Tropical Research Ethics Committee, University of Oxford (UK, 025-04). All participants provided written informed consent.

### Assessment of alcohol consumption

Detailed questionnaire assessment of alcohol consumption has been described previously^3,17,56^. In the baseline questionnaire, participants were asked how often they had drunk alcohol during the past 12 months (never or almost never, occasionally, only at certain seasons, every month but less than weekly or usually at least once a week). Those who had not drunk alcohol at least weekly in the past 12 months were asked whether there was a period of at least a year before that when they had drunk some alcohol at least once a week. Based on their past and current drinking history, participants were classified into: non-drinkers (had never drunk alcohol in the past year and had not drunk in most weeks in the past); ex-drinkers (had not drunk alcohol in most weeks in the past year but had done so in the past); occasional drinkers (had drunk alcohol but less than weekly in the past year and had not drunk alcohol in most weeks in the past); and current drinkers (had drunk alcohol on a weekly basis (regularly) in the past year).

Current drinkers were asked further questions about their drinking patterns, including frequency, beverage type (beer, grape wine, rice wine, weak spirits with <40% alcohol content and strong spirits with ≥40% alcohol content) and amount consumed on a typical drinking day, mealtime drinking habits, age started drinking in most week and their experience of flushing or dizziness after drinking.

Alcohol intake level was estimated based on the reported frequency (taken as the median of the reported frequency intervals; 1.5 for 1–2 d per week, 4 for 3–5 d per week, 6.5 for 6–7 d per week), beverage type and amount consumed, assuming the following alcohol content by volume (v/v) typically seen in China: beer 4%, grape wine 12%, rice wine 15%, weak spirits 38% and strong spirits 53%^57^. Among current drinkers, men were grouped into four consumption categories (<140, 140–279, 280–419 and 420+ g per week) and women into three categories (<70, 70–139 and 140+ g per week), broadly based on the recommended cutoffs for alcohol categories by the WHO^58^ and national drinking guidelines. Heavy episodic drinking was defined as consuming >60 g of alcohol on a typical drinking occasion for men and >40 g per occasion for women^58^. Drinking outside of meals was defined as usually drinking between or after meals or having no regular patterns (versus usually drinking with meals). Duration of drinking was derived by the difference in years between age at baseline and age started drinking.

Ex-drinkers were asked how long (in years) ago they had stopped drinking in most weeks. Ex-drinkers were grouped with current drinkers as ‘ever-regular drinkers’.

### Follow-up for mortality and morbidity

The vital status of participants was obtained periodically from local death registries, supplemented by annual active confirmation through local residential, health insurance and administrative records. Additional information on morbidity was collected through linkage with disease registries (for cancer, stroke, IHD and diabetes) and the national health insurance system, which record any episodes of hospitalization and almost has universal coverage. All events were coded with ICD-10 codes, blinded to the baseline information. By 1 January 2019, 56,550 (11%) participants had died, 311,338 (61%) were ever hospitalized, but only 4,028 (<1%) were lost to follow-up.

### Outcome measures

To enable a ‘phenome-wide’ investigation, all recorded diseases and injuries (referred to as ‘diseases’ for simplicity) coded by three-character ICD-10 codes were reviewed. ICD-10 codes were combined (where appropriate) based on disease characteristics and their potential relationships with alcohol consumption^4,8,10,59^. Disease end points were curated based on diseases considered to be causally impacted by alcohol by the WHO^4,59^ and major diseases previously shown to be related to alcohol in CKB and other large prospective cohort studies^8,10^, while retaining maximal granularity. Diseases with at least 80 cases recorded during follow-up among current drinkers, separately by sex, were analyzed individually to capture a wide range of specific conditions while ensuring reasonable statistical power (around 60–80% power to detect a HR of 2.00 per 280 g per week higher usual alcohol intake at *P* < 0.01 and *P* < 0.05, respectively). Within each ICD-10 chapter, diseases with <80 events were grouped into a ‘less-common’ category. Several ICD-10 chapters considered not directly relevant in this population (for example perinatal-origin diseases (chapter XVI) and congenital conditions (XVII); pregnancy-related diseases (XV) in men) were excluded.

Major diseases defined by the WHO as likely to be causally related with alcohol consumption^4^, including several cancers (mouth and throat, esophagus, colon-rectum, liver and female breast), diabetes mellitus, IHD, stroke, liver cirrhosis and external causes, were also selected a priori for detailed analyses of associations with drinking patterns (daily drinking, heavy episodic drinking, mealtime habit, spirit drinking and drinking duration). Similarly, diseases that were significantly and adversely associated with alcohol in the ‘phenome-wide’ investigations (either with ever-regular versus occasional drinking or in dose–response associations with amounts consumed) were further categorized as ‘CKB WHO alcohol-related diseases’ and ‘CKB new alcohol-associated diseases’ respectively for genetic investigation of causality. Detailed outcome classifications are reported in Supplementary Table 7.

### Genotyping and alcohol genetic instruments

The two East Asian genetic variants (*ALDH2*-rs671 and *ADH1B*-rs1229984) were genotyped in 168,050 participants (151,347 randomly selected, 16,703 selected as part of nested case–control studies of CVD and chronic obstructive pulmonary disease, which were only included in analyses of relevant outcomes; Supplementary Fig. 1) using Affymetrix Axiom (*n* = 100,396) or custom Illumina GoldenGate (*n* = 93,125) arrays at BGI (Shenzhen, China), with some overlap between them. Among 25,471 participants genotyped with both arrays, the concordance was >99.9% for both variants. Where discordant, genotypes obtained from the Affymetrix Axiom array were used.

The genetic instrument for alcohol was derived from *ALDH2*-rs671 and *ADH1B*-rs1229984 and ten study areas from the random genotyped subset of male participants to avoid potential selection bias, using a previously developed method in CKB^19^. Briefly, nine genotype combinations were defined based on the genotypes for each of the two variants (each AA, AG or GG). As alcohol use varies greatly by study area, among men, mean alcohol intake was calculated for each of these nine genotype across ten study areas (that is a total of 90 genotype-area combinations) to reflect a wide range of alcohol consumption, assigning an intake of 5 g per week to occasional drinkers and excluding ex-drinkers from the calculation. Ex-drinkers were excluded from the calculation of mean alcohol intake as their baseline intake did not reflect their long-term intake; nevertheless, they were included in subsequent genetic analyses once they had been assigned a genetic group. These 90 combinations were then grouped into six categories (C1–C6) according to their corresponding mean intake values, at cutoff points of 10, 25, 50, 100 and 150 g per week, selected to facilitate investigation of the causal effects of alcohol across a wide range of mean alcohol intakes while allowing adequate sample size in each category for reliable comparisons. In this way participants (including ex-drinkers) were classified only based on their genotypes and study area, but not on individual self-reported drinking patterns. Comparisons of these six genetic categories can, where analyses are stratified by area, be used to estimate the genotypic effects on disease risks.

To facilitate the comparison of genotypic effects between sexes (pleiotropic effects), women were classified into the same six categories as men based on their genotypes and study area, regardless of female alcohol intake. This allowed comparison of genotypic effects between men (where genotype were strongly associated with alcohol intake) and women (where alcohol intake was low in all genotypic categories) (Supplementary Tables 10 and 11).

### Statistical analysis

Given the extremely low alcohol use among women^3,17^, the analyses were conducted separately by sex but focused chiefly on men. All CKB participants and the genotyped subset with genomic principal components (PCs; derived from genome-wide genotyping array data and were informative for CKB population structure)^60^ were included in conventional and genetic analyses, respectively (Supplementary Fig. 1). Means and percentages of baseline characteristics were calculated by self-reported alcohol consumption patterns and by genotype categories, adjusted for age (in 10-year intervals), ten study areas and (for genetic analysis) genomic PCs^60^ to control for differences in genetic distribution due to population stratification, as appropriate.

For conventional observational analyses, Cox proportional hazard models were used to estimate HRs for individual diseases associated with different alcohol consumption categories (in three broad categories: occasional drinkers, ever-regular drinkers, non-drinkers; and in 6–7 detailed categories: occasional drinkers, ex-drinkers, non-drinkers, 3–4 further current drinker groups defined by alcohol intake level) and among current drinkers with continuous levels of alcohol intake (per 280 g per week in men, per 100 g per week in women) or with categories of alcohol intake (<140, 140–279, 280–419 and 420+ g per week in men; <70, 70–139 and 140+ g per week in women). The Cox models were stratified by age at risk (5-year groups between 35–84 years) and ten areas and adjusted for education (four groups: no formal school, primary school, middle or high school and technical school/college or above) and smoking status (six groups in men: never, occasional, ex-regular, current <15, current 15–24, current ≥25 cigarettes equivalent per day; four groups in women: never, occasional, ex-regular and current). Smoking data have been previously validated against exhaled carbon monoxide^61^. Competing risks from all-cause mortality for disease events were handled by censoring participants at death from any cause to estimate cause-specific HRs comparing event rates in participants who were alive and free of the disease of interest^62^. To reduce biases from residual confounding and uncontrolled reverse causation related to the choice of using non-drinkers (for example sick-quitter effect, pre-existing poor health or social disadvantages leading to alcohol cessation or abstinence) as the reference group^3,29^, we used occasional drinkers as the reference group, together with separate dose–response analyses among current drinkers. To account for within-person variation of alcohol intake over the follow-up period, repeat alcohol measures for participants who attended the two resurveys were used to estimate usual alcohol intake (Supplementary Table 1) and correct for regression dilution bias^9,63^. The shapes of dose–response associations between alcohol and disease risks were assessed among current drinkers by plotting the HRs of predefined baseline consumption categories against the corresponding mean usual alcohol intake. Log HR estimates and the corresponding standard errors for baseline alcohol intake, modeled as a continuous variable, were divided by the regression dilution ratio (0.53 for both men and women; calculated using the McMahon–Peto method^64^) to obtain estimated HRs per 280 g per week higher usual alcohol intake among male current drinkers and HRs per 100 g per week among female current drinkers. For analyses involving drinking patterns, additional adjustments were conducted for total alcohol intake (continuous) and baseline age (continuous; for drinking duration analysis) where appropriate.

Sensitivity analyses were performed by (1) additional adjustments for further covariates (household income (<10,000, 10,000–19,999, 20,000–34,999 and ≥35,000 yuan per year), fresh fruit intake (4–7 d per week and ≤3 d per week), physical activity (continuous, in metabolic equivalent of task per hour per day), body mass index (<22, 22–24.9, 25–26.9 and ≥27 kg m^2^); and (2) excluding individuals with poor self-reported health or previous major chronic diseases (including self-reported coronary heart diseases, stroke, transient ischemic attack, tuberculosis, emphysema or bronchitis, liver cirrhosis or chronic hepatitis, peptic ulcer, gallstone or gallbladder disease, kidney disease, rheumatoid arthritis, cancer and diabetes) at baseline. For all aggregated end points (for example CKB WHO alcohol-related, CKB new alcohol-associated and all morbidity), subgroup analyses were conducted by baseline age (<55, 55–64 and ≥65 years), area (urban and rural), education (primary school or below, middle school, high school or above), household income (<10,000, 10,000–19,999 and 20,000+ yuan per year) and smoking status (ever-regular and never-regular), with heterogeneity or trend assessed by chi-squared tests^65^. HRs for diseases associated with years of stopping among ex-drinkers compared to occasional drinkers were also estimated.

In genetic analyses, Cox regression, stratified by age at risk and study area and adjusted for 11 genomic PCs^60^, were used to estimate HRs for major alcohol-related diseases associated with the six genetic categories (C1–C6). Log HRs were plotted against the genotype-predicted mean male alcohol intake in the six categories. To control for potential confounding by population structure, similar analyses were repeated within each study area using age-at-risk-stratified and genomic PC-adjusted Cox models. A line of best fit was fitted through the log HRs against genotype-predicted mean male alcohol intake in the genetic categories present in the corresponding study area, using meta-regression. These within-area slopes (each reflecting purely genotypic effects) were combined by inverse-variance-weighted meta-analysis to yield the overall area-stratified genotypic associations, which controlled for any potential bias resulted from variations due to population structure, summarized as HR per 280 g per week higher genotype-predicted mean male alcohol intake. For total morbidity and aggregated alcohol-associated outcomes, sensitivity analyses were performed by (1) using age-at-risk- and area-stratified and genomic PC-adjusted Cox models to estimate HR per 280 g per week (area-adjusted genotypic associations); and (2) using a two-stage least-squares approach^66^.

Genotypic analyses in women were conducted not to assess the health effects of alcohol in women, but to investigate the extent to which the genotypes studied in men had pleiotropic effects (genotypic effects not mediated by drinking patterns). As few women consumed alcohol, any genotypic effects of the six genetic categories that are mediated by drinking alcohol should be much smaller in women than in men, but any other pleiotropic genotypic effects should be similar in both sexes. Hence, among women, we used the same genetic categories as in men and related the genotypic effects in women to the mean male alcohol intake in these six categories, which allows comparisons of genetic findings by sex and assessment of potential pleiotropy. To further remove the small genotypic effects on alcohol use in women (Supplementary Tables 10 and 11), we restricted the genetic analyses to female non-drinkers in sensitivity analyses.

The genotypic associations of individual genetic variants (rs671, rs1229984; GG versus AG genotype) with alcohol-related disease risks were also assessed using a similar area-stratified approach.

The proportional hazards assumption was tested using scaled Schoenfeld residuals for the pre-specified major diseases (no clear evidence of violation was found). For analyses involving more than two exposure categories, the floating absolute risks were used to estimate group-specific 95% CIs for all categories including the reference group^9,19,67^. All *P* values were two-sided. Statistical significance (at the 5% level) was evaluated using both FDR-adjusted *P* values applied within ICD-10 chapters to correct for multiple testing in the ‘phenome-wide’ investigation^68–70^ and conventional *P* values for hypothesis testing for observational analyses of WHO alcohol-related diseases, analyses of drinking patterns and genetic analyses.

To assess the cumulative burden of alcohol consumption, the total number of hospitalizations were estimated for ever-regular versus occasional drinkers using the mean cumulative count, which does not assume independence between hospitalizations and all-cause mortality^71–73^. All analyses used R software (v.4.0.5).

### Ethics and inclusion statement

In accordance with the Nature Portfolio journals’ editorial policies, the research has included local researchers from China throughout the research process, including study design, study implementation, data ownership and authorship. The roles and responsibilities were agreed among collaborators ahead of the research and capacity-building plans, including data collection and study implementation skills for local researchers, were discussed and delivered. This research is locally relevant to the studied country and included local collaborative partners in all aspects of the study, thus, will provide local and regional organizations with epidemiological evidence on the health impacts of alcohol consumption to inform public health policies.

This research was not restricted nor prohibited in the setting of the researchers. The study was approved by local ethics review committee. The research raised no risks related to stigmatization, incrimination, discrimination, animal welfare, the environment, health, safety, security or other personal or biorisks. No biological materials, cultural artifacts or associated traditional knowledge has been transferred out of the country. In preparing the manuscript, the authors have reviewed and cited local and regional relevant studies.

### Reporting summary

Further information on research design is available in the Nature Portfolio Reporting Summary linked to this article.

## Online content

Any methods, additional references, Nature Portfolio reporting summaries, source data, extended data, supplementary information, acknowledgements, peer review information; details of author contributions and competing interests; and statements of data and code availability are available at 10.1038/s41591-023-02383-8.

## Supplementary information

Supplementary Information Supplementary Figs. 1–8 and Supplementary Tables 1–13.

Reporting Summary

## Data Availability

The CKB is a global resource for the investigation of lifestyle, environmental, blood biochemical and genetic factors as determinants of common diseases. The CKB study group is committed to making the cohort data available to the scientific community in China, the United Kingdom and worldwide to advance knowledge about the causes, prevention and treatment of disease. For detailed information on what data are currently available to open access users, how to apply for them and the timeline for data access (12–16 weeks), please visit the CKB website: https://www.ckbiobank.org/data-access. Researchers who are interested in obtaining the raw data from the CKB study that underlines this paper should contact ckbaccess@ndph.ox.ac.uk. A research proposal will be requested to ensure that any analysis is performed by bona fide researchers and, where data are not currently available to open access researchers, is restricted to the topic covered in this paper. Further information is available from the corresponding authors upon request.

## Code availability

The codes used for the data analyses in this study can be made available by contacting the corresponding authors. Access to codes will be granted for requests for academic use within 4 weeks of application.
