# Supplementary material for: Alcohol consumption and risks of more than 200 diseases in Chinese men
Source: Nat Med. 2023 Jun 8;29(6):1476–86. doi: 10.1038/s41591-023-02383-8 (PMC10287564; doi:10.1038/s41591-023-02383-8)
Supplement: Supplementary file 1 — Supplementary Figs. 1–8 and Supplementary Tables 1–13. [file 41591_2023_2383_MOESM1_ESM.pdf]

nature medicine

Article

https://doi.org/10.1038/s41591-023-02383-8

Alcohol consumption and risks of more than 200 diseases in Chinese men

In the format provided by the authors and unedited

Supplementary material

Alcohol consumption and risks of more than 200 diseases in Chinese men

Pek Kei Im, Neil Wright, Ling Yang, Ka Hung Chan, Yiping Chen, Yu Guo, Huaidong Du, Xiaoming Yang, Daniel Avery, Shaojie Wang, Canqing Yu, Jun Lv, Robert Clarke, Junshi Chen, Rory Collins, Robin G Walters, Richard Peto, Liming Li, Zhengming Chen, Iona Y Millwood, China Kadoorie Biobank (CKB) Collaborative Group

Content List of Supplementary Material

Members of the China Kadoorie Biobank collaborative group 3

Supplementary Figure 1. Study participant flowchart 4

Supplementary Table 1. Self-reported alcohol intake at baseline and resurveys, by baseline alcohol grouping 5

Supplementary Table 2. Baseline characteristics by drinking patterns, in male current drinkers 6

Supplementary Table 3. Mean duration of drinking by alcohol intake categories, in male and female current drinkers 7

Supplementary Table 4. Baseline characteristics by drinking patterns, in female current drinkers 8

Supplementary Table 5. Summary of number of diseases associated with alcohol consumption by ICD-10 chapter, in women 9

Supplementary Table 6. Adjusted HRs for overall and specific morbidities associated with alcohol drinking status, by ICD-10 chapter in men 10

Supplementary Table 7. Outcome classifications of pre-specified major diseases and aggregate endpoints 14

Supplementary Figure 2. Adjusted HRs for ICD-10 Chapters I to VIII associated with usual alcohol intake, in male current drinkers 15

Supplementary Figure 3. Adjusted HRs for ICD-10 Chapters IX to XI associated with usual alcohol intake, in male current drinkers 16

Supplementary Figure 4. Adjusted HRs for ICD-10 Chapters XII to XX associated with usual alcohol intake, in male current drinkers 17

Supplementary Figure 5. Adjusted HRs per 280 g/week higher usual alcohol intake for specific alcohol-associated diseases in male current drinkers, with further adjustments or exclusion of participants with baseline medical conditions 18

Supplementary Table 8. Adjusted HRs per 280 g/week higher usual alcohol intake for major diseases in male current drinkers, with further adjustments or exclusion of participants with baseline medical conditions 19

Supplementary Figure 6. Adjusted HRs for CKB WHO alcohol-related diseases, CKB new alcohol-associated diseases and all morbidity per 280 g/week higher usual alcohol intake, by population subgroups in male current drinkers 20

Supplementary Figure 7. Associations of aggregate disease categories with self-reported alcohol intake and with genotype-predicted mean male alcohol intake categories, in women 21

Supplementary Table 9. Genotype distribution and allele frequencies of *ALDH2*-rs671 and *ADH1B*-rs1229984 across the ten study areas 22

Supplementary Table 10. Baseline characteristics and alcohol drinking patterns by *ALDH2*-rs671 genotype, and by *ADH1B*-rs1229984 genotype 23

Supplementary Table 11. Baseline characteristics and alcohol drinking patterns by six genetic categories (C1-C6) 24

Supplementary Table 12. Adjusted HRs per 280 g/week higher genotype-predicted alcohol intake with alcohol-related diseases and overall morbidity, sensitivity analyses in men 25

Supplementary Figure 8. Total expected hospitalisations overall and by major disease categories in ever- and never-regular drinkers from age-at-risk of 35 years among men......26
Supplementary Table 13. ICD-10 code mapping of CKB significant WHO alcohol-related diseases to alcohol-related diseases based on WHO-classifications......27

Members of the China Kadoorie Biobank collaborative group

International Steering Committee: Junshi Chen, Zhengming Chen (PI), Robert Clarke, Rory Collins, Yu Guo, Liming Li (PI), Chen Wang, Jun Lv, Richard Peto, Robin Walters.

International Co-ordinating Centre, Oxford: Daniel Avery, Maxim Barnard, Derrick Bennett, Ruth Boxall, Ka Hung Chan, Yiping Chen, Zhengming Chen, Johnathan Clarke; Robert Clarke, Huaidong Du, Ahmed Edris Mohamed, Hannah Fry, Simon Gilbert, Pek Kei Im, Andri Iona, Maria Kakkoura, Christiana Kartsonaki, Hubert Lam, Kuang Lin, James Liu, Mohsen Mazidi, Iona Millwood, Sam Morris, Qunhua Nie, Alfred Pozarickij, Paul Ryder, Saredo Said, Dan Schmidt, Becky Stevens, Iain Turnbull, Robin Walters, Baihan Wang, Lin Wang, Neil Wright, Ling Yang, Xiaoming Yang, Pang Yao.

National Co-ordinating Centre, Beijing: Xiao Han, Can Hou, Qingmei Xia, Chao Liu, Jun Lv, Pei Pei, Yu Guo, Dianjianyi Sun, Canqing Yu.

10 Regional Co-ordinating Centres:

Guangxi Provincial CDC: Naying Chen, Duo Liu, Zhenzhu Tang. Liuzhou CDC: Ningyu Chen, Qilian Jiang, Jian Lan, Mingqiang Li, Yun Liu, Fanwen Meng, Jinhuai Meng, Rong Pan, Yulu Qin, Ping Wang, Sisi Wang, Liuping Wei, Liyuan Zhou. **Gansu** Provincial CDC: Caixia Dong, Pengfei Ge, Xiaolan Ren. Maiji CDC: Zhongxiao Li, Enke Mao, Tao Wang, Hui Zhang, Xi Zhang. **Hainan** Provincial CDC: Jinyan Chen, Ximin Hu, Xiaohuan Wang. Meilan CDC: Zhendong Guo, Huimei Li, Yilei Li, Min Weng, Shukuan Wu. **Heilongjiang** Provincial CDC: Shichun Yan, Mingyuan Zou, Xue Zhou. Nangang CDC: Ziyan Guo, Quan Kang, Yanjie Li, Bo Yu, Qinai Xu. **Henan** Provincial CDC: Liang Chang, Lei Fan, Shixian Feng, Ding Zhang, Gang Zhou. Huixian CDC: Yulian Gao, Tianyou He, Pan He, Chen Hu, Huarong Sun, Xukui Zhang. **Hunan** Provincial CDC: Biyun Chen, Zhongxi Fu, Yuelong Huang, Huilin Liu, Qiaohua Xu, Li Yin. Liuyang CDC: Huajun Long, Xin Xu, Hao Zhang, Libo Zhang. **Jiangsu** Provincial CDC: Jian Su, Ran Tao, Ming Wu, Jie Yang, Jinyi Zhou, Yonglin Zhou. Suzhou CDC: Yihe Hu, Yujie Hua, Jianrong Jin, Fang Liu, Jingchao Liu, Yan Lu, Liangcai Ma, Aiyu Tang, Jun Zhang. **Qingdao** CDC: Liang Cheng, Ranran Du, Ruqin Gao, Feifei Li, Shanpeng Li, Yongmei Liu, Feng Ning, Zengchang Pang, Xiaohui Sun, Xiaocao Tian, Shaojie Wang, Yaoming Zhai, Hua Zhang, Licang CDC: Wei Hou, Silu Lv, Junzheng Wang. **Sichuan** Provincial CDC: Xiaofang Chen, Xianping Wu, Ningmei Zhang, Xiaoyu Chang. Pengzhou CDC: Xiaofang Chen, Jianguo Li, Jiaqiu Liu, Guojin Luo, Qiang Sun, Xunfu Zhong. **Zhejiang** Provincial CDC: Weiwei Gong, Ruying Hu, Hao Wang, Meng Wang, Min Yu. Tongxiang CDC: Lingli Chen, Qijun Gu, Dongxia Pan, Chunmei Wang, Kaixu Xie, Xiaoyi Zhang.

Supplementary Figure 1. Study participant flowchart

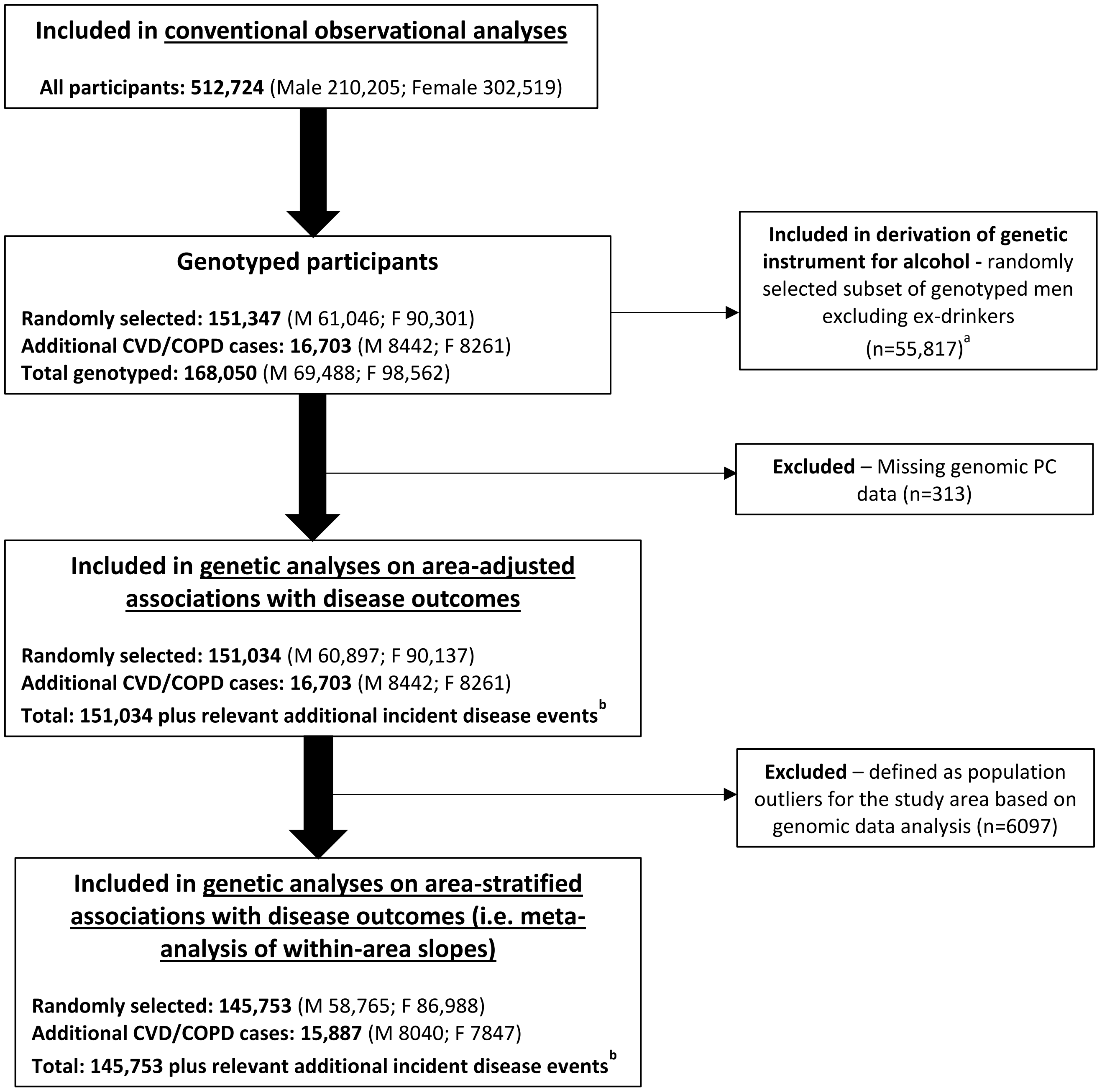

[a] The genetic instrument (six genetic categories C1-C6) was derived from the randomly selected subset of genotyped men excluding ex-drinkers (n=61,046 randomly selected, of whom n=5229 were ex-drinkers). The six genetic categories was then assigned to all genotyped men and women according to their genotype and area, regardless of individual drinking patterns.
[b] To increase study power, additional recorded disease events (mainly but not limited to CVD/COPD) recorded during follow-up were selected from the subset of CVD/COPD cases and added to the genetic analyses of the corresponding disease outcome.
CVD, cardiovascular disease; COPD, chronic obstructive pulmonary disease; PC, principal components.

Supplementary Table 1. Self-reported alcohol intake at baseline and resurveys, by baseline alcohol grouping

Baseline alcohol grouping	Baseline (2004-2008) N	Baseline (2004-2008) Mean intake, g/week	1st resurvey (2008) N	1st resurvey (2008) Mean intake, g/week	2nd resurvey (2013-2014) N	2nd resurvey (2013-2014) Mean intake, g/week	Average of 2 resurveys Mean usual intake, g/week	Regression dilution ratio[a]
**Men**								
Non-drinkers	42779	0.0	1548	3.6	1845	4.2	3.9	
Ex-drinkers	18295	2.8	652	37.8	767	67.0	52.4	
Occasional drinkers	79231	5.0	2960	21.0	3659	33.6	27.3	
Current drinkers								
<140 g/week	25093	79.5	987	97.1	1199	119.4	108.2	
140-279 g/week	18907	222.6	659	185.0	888	233.1	209.1	
280-419 g/week	12832	370.2	463	283.9	589	306.8	295.4	
420+ g/week	13068	689.7	503	440.8	632	418.0	429.4	0.53
**Women**								
Non-drinkers	192333	0.0	7602	0.8	9988	0.7	0.8	
Ex-drinkers	2657	2.5	113	13.4	151	11.8	12.6	
Occasional drinkers	101285	5.0	4036	4.6	4974	3.8	4.2	
Current drinkers								
<70 g/week	3224	34.0	124	27.9	162	21.1	24.5	
70-139 g/week	1587	112.5	69	89.9	93	66.0	77.9	
140+ g/week	1433	302.5	70	199.4	94	136.5	167.9	0.53

Calculations assigned an intake of 5 g/week to those who drink sometimes but less than weekly at the time of survey (regardless of their past drinking patterns).
[a] Regression dilution ratio is estimated among baseline current drinkers using baseline and usual alcohol intakes. It was calculated using the assumption-free, non-parametric McMahon-Peto method i.e. the ratio of the range of the usual alcohol intake levels to the range of the baseline alcohol intake levels of baseline-defined current drinker groups.

Supplementary Table 2. Baseline characteristics by drinking patterns, in male current drinkers

	All current drinkers	Drinking days per week		Heavy episodic drinking (> 60 g/session)		Mealtime drinking		Beverage type		
		1-5 days	6-7 days	No	Yes	With meals	Outside of meals	Beer	Wine	Spirits
	(N=69900)	(N=26521)	(N=43379)	(N=43891)	(N=26009)	(N=60043)	(N=9857)	(N=12723)	(N=8506)	(N=48671)
**Sociodemographic characteristics**										
Mean age, years	51.6	48.5	53.5	52.6	49.7	51.6	51.2	45.4	56.0	52.5
Urban, %	50.1	52.6	48.0	57.0	37.6	52.0	38.8	83.4	72.4	37.4
Education > 6 years, %	60.3	64.3	58.2	61.0	58.8	60.5	59.9	66.3	63.2	59.8
Household income > 20,000 yuan/year, %	51.5	54.4	49.4	51.2	51.9	52.0	48.6	59.7	59.8	50.7
**Alcohol drinking patterns**										
Mean weekly intake, g/week	285.7	123.4	377.7	163.1	494.0	282.2	319.7	131.1	117.3	329.2
Daily drinking, %	62.1	--	--	56.7	69.8	61.7	63.4	43.3	57.3	66.2
Heavy episodic drinking, %	37.2	25.3	41.2	--	--	36.7	40.0	11.9	3.4	45.0
Drinking outside of meals, %	14.1	13.4	14.5	13.5	15.1	--	--	12.2	10.6	14.1
Drinking spirits, %	69.6	62.6	73.8	62.6	85.5	69.6	69.0	--	--	--
Flushing response, %	17.9	22.4	15.5	22.3	11.6	17.9	18.3	22.9	16.8	17.3
Duration of drinking, years	23.0	21.9	23.5	22.0	24.9	22.9	23.8	21.6	20.3	23.3
Age started drinking, years	28.7	29.5	28.3	29.7	26.5	28.7	27.2	29.6	29.9	28.4
**Lifestyle risk factors**										
Current smokers, %	71.3	67.3	73.8	68.0	76.8	71.2	71.0	62.9	63.4	74.0
Infrequent fresh fruit intake, %	77.2	73.8	79.4	75.2	81.1	77.1	76.7	68.2	56.8	79.0
Physical activity, mean MET-h/d	22.9	22.7	23.0	23.1	22.7	22.9	22.9	21.6	20.2	23.2
Mean systolic blood pressure, mmHg	134.2	132.7	135.1	133.0	136.4	134.3	132.8	129.5	127.9	135.2
Mean body mass index, kg/m^2	23.7	24.0	23.5	23.6	24.0	23.7	23.3	23.8	23.4	23.7
**Self-reported medical history, %**										
Poor self-reported health	6.3	6.6	6.4	6.4	6.5	6.2	6.9	5.8	8.0	6.3
Prior chronic disease[a]	18.2	19.7	17.7	18.5	17.5	18.1	19.2	19.9	24.4	17.5

Means and percentages are adjusted for the age and study area structure of the CKB male current drinker population, using direct standardisation.
[a] Chronic diseases included self-reported history of coronary heart disease, stroke, transient ischaemic attack, diabetes, tuberculosis, emphysema/chronic bronchitis, liver cirrhosis/chronic hepatitis, peptic ulcer, gallstone/gallbladder disease, kidney disease, rheumatoid arthritis, and cancer.

Supplementary Table 3. Mean duration of drinking by alcohol intake categories, in male and female current drinkers

	< 140 (men) / < 70 (women) g per week	140-279 (men) / 70-139 (women) g per week	280-419 (men) / ≥ 140 (women) g per week	≥ 420 (men) g per week	P value for trend
**Men**					
Adjusted mean duration of drinking (95% CI), years	20.9 (20.8-21.0)	23.0 (22.8-23.1)	24.3 (24.1-24.4)	25.6 (25.5-25.8)	$< 1 \times 10^{-10}$
**Women**					
Adjusted mean duration of drinking (95% CI), years	13.6 (13.1-14.0)	16.0 (15.5-16.6)	19.0 (18.4-19.6)	--	$< 1 \times 10^{-10}$

Adjusted means and 95% CIs were estimated using multiple linear regression, adjusted for age (in ten-year intervals) and ten study areas, in male and female current drinkers respectively. P values for trend were obtained from multiple linear regression models assessing the dose-response per g/week increase in alcohol intake. All P values are two-sided. CI, confidence interval.

Supplementary Table 4. Baseline characteristics by drinking patterns, in female current drinkers

	All current drinkers	Drinking days per week		Heavy episodic drinking (> 40 g/session)		Mealtime drinking		Beverage type		
		1-5 days	6-7 days	No	Yes	With meals	Outside of meals	Beer	Wine	Spirits
	(N=6244)	(N=3418)	(N=2826)	(N=5656)	(N=588)	(N=5384)	(N=860)	(N=1387)	(N=1001)	(N=3856)
**Sociodemographic characteristics**										
Mean age, years	53.2	51.2	56.2	53.4	49.5	53.0	55.8	46.5	55.4	54.5
Urban, %	45.1	53.3	32.7	46.7	27.5	43.7	53.6	89.1	77.5	21.3
Education > 6 years, %	47.5	49.1	46.3	47.8	42.1	47.9	41.4	54.4	58.2	44.6
Household income > 20,000 yuan/year, %	39.4	40.6	38.0	39.2	34.1	40.1	40.9	42.0	66.8	36.8
**Alcohol drinking patterns**										
Mean weekly intake, g/week	115.6	58.9	168.9	90.7	328.8	115.7	92.1	49.2	36.2	138.9
Daily drinking, %	45.3	--	--	43.9	49.4	44.0	43.2	20.8	37.2	48.6
Heavy episodic drinking, %	9.4	5.8	10.6	--	--	9.4	6.7	2.9	0.1	12.0
Drinking outside of meals, %	13.8	12.3	15.4	13.7	11.6	--	--	6.9	13.7	14.0
Drinking spirits, %	61.8	59.3	65.0	60.9	76.3	61.5	61.2	--	--	--
Flushing response, %	23.6	23.9	23.5	24.5	15.4	23.3	26.5	24.9	24.3	24.0
Duration of drinking, years	15.4	15.1	15.4	15.1	17.9	15.8	12.4	13.9	14.5	15.8
Age started drinking, years	37.7	37.8	37.9	38.1	30.5	37.3	40.1	34.2	36.0	37.4
**Lifestyle risk factors**										
Current smokers, %	15.7	12.0	18.2	14.4	24.1	15.8	9.5	7.1	5.6	17.8
Infrequent fresh fruit intake, %	59.1	58.0	60.1	58.8	62.0	58.8	64.1	50.8	24.4	62.6
Physical activity, mean MET-h/d	20.0	20.1	19.8	20.0	18.7	20.1	17.3	18.0	15.8	20.0
Mean systolic blood pressure, mmHg	128.2	127.8	128.4	128.0	121.8	128.1	122.8	116.7	114.4	128.6
Mean body mass index, kg/m^2	23.7	23.9	23.6	23.7	22.3	23.7	22.9	22.5	22.4	23.8
**Self-reported medical history, %**										
Poor self-reported health	10.2	10.0	10.9	10.1	8.7	9.9	5.9	9.2	4.9	10.6
Prior chronic disease[a]	22.2	23.1	23.2	22.3	17.5	21.8	27.3	24.2	23.8	22.7

Means and percentages are adjusted for the age and study area structure of the CKB female current drinker population, using direct standardisation.
[a] Chronic diseases included self-reported history of coronary heart disease, stroke, transient ischaemic attack, diabetes, tuberculosis, emphysema/chronic bronchitis, liver cirrhosis/chronic hepatitis, peptic ulcer, gallstone/gallbladder disease, kidney disease, rheumatoid arthritis, and cancer.

Supplementary Table 5. Summary of number of diseases associated with alcohol consumption by ICD-10 chapter, in women

ICD-10 Chapter	No of diseases	Total No of events	No of events in current drinkers	Ever-regular vs. occasional drinking		Dose-response associations among current drinkers		Total[a]	
				Positive	Negative	Positive	Negative	Positive	Negative
I Infectious and parasitic	2	10858	283	0	0	0	0	0	0
II Neoplasms	2	29510	619	0	0	0	0	0	0
III Blood and immune-related	1	2831	71	0	0	0	0	0	0
IV Endocrine, nutritional and metabolic	3	28424	625	0	0	0	0	0	0
V Psychiatric and behavioural	1	3556	90	0	0	0	0	0	0
VI Nerve-related	2	12518	282	0	0	0	0	0	0
VII Eye and adnexa	2	18012	380	0	0	0	0	0	0
VIII Ear and mastoid process	2	5099	125	0	0	0	0	0	0
IX Circulatory	7	135578	3136	2	0	0	0	2	0
X Respiratory	5	51675	1537	0	1	0	0	0	1
XI Digestive	7	52823	1438	0	0	0	0	0	0
XII Skin and subcutaneous tissue	1	2329	80	0	0	0	0	0	0
XIII Musculoskeletal	5	47022	1224	0	1	0	0	0	1
XIV Genitourinary	2	27202	589	0	0	0	0	0	0
XV Pregnancy-related	1	1299	18	0	0	0	0	0	0
XVIII Other symptoms, signs and abnormal findings	3	28829	780	0	0	0	0	0	0
XIX Injury, poisoning and other external causes	1	17732	472	0	0	0	0	0	0
XX External causes	1	1689	24	0	0	0	0	0	0
**Total**	**48**	**476986**	**11773**	**2**	**2**	**0**	**0**	**2**[b]	**2**[c]

ICD-10, International Classification of Diseases, 10th Revision; WHO, World Health Organization; FDR, false discovery rate.

[a] Included disease associations from Cox regression analyses either significant (p<0.05 for diseases classified as alcohol-related by the WHO, FDR-adjusted p<0.05 for other diseases; two-sided) from the comparison of ever-regular vs. occasional drinking or dose-response association analyses within current drinkers.

[b] The 2 diseases were intracerebral haemorrhage (ICD-10 code: I61) (HR 1.20, 95% CI 1.03-1.40) and essential primary hypertension (I10) (HR 1.08, 95% CI 1.01-1.16), both of which were classified as alcohol-related by the WHO and showed nominally significant associations with ever-regular drinking (i.e. P value <0.05).

[c] The 2 diseases were other acute lower respiratory infections (J20-J22) (HR 0.88, 95% CI 0.78-0.99) which was classified as alcohol-related by the WHO and showed nominal association with ever-regular drinking, and spondylosis (M47) (HR 0.87, 95% CI 0.80-0.95) which was significantly associated with ever-regular drinking after multiple testing correction (i.e. FDR-adjusted P value < 0.05).

Supplementary Table 6. Adjusted HRs for overall and specific morbidities associated with alcohol drinking status, by ICD-10 chapter in men

	Overall	Ex-drinkers		Non-drinkers		Occasional drinkers		Current drinkers							
	N of							<140 g/week		140-279 g/week		280-419 g/week		420+ g/week	
ICD-10 coded disease	events	N	HR (95% CI)	N	HR (95% CI)	N	HR (95% CI)	N	HR (95% CI)	N	HR (95% CI)	N	HR (95% CI)	N	HR (95% CI)
**I Infectious and parasitic**															
A15-A19, B90 Tuberculosis	1794	222	1.35 (1.18-1.54)	442	1.27 (1.15-1.40)	575	1.00 (0.92-1.09)	207	1.02 (0.89-1.17)	146	1.02 (0.87-1.21)	85	1.02 (0.82-1.27)	117	1.51 (1.25-1.83)
Any	7647	975	1.30 (1.22-1.39)	1791	1.20 (1.14-1.26)	2476	1.00 (0.96-1.04)	849	1.00 (0.93-1.07)	600	0.92 (0.85-1.00)	416	0.98 (0.89-1.08)	540	1.16 (1.06-1.27)
**II Neoplasms**															
C00-C14 Lip, oral cavity & pharynx cancer	531	64	1.34 (1.05-1.72)	98	0.95 (0.77-1.17)	168	1.00 (0.85-1.18)	38	0.68 (0.49-0.93)	63	1.42 (1.11-1.82)	33	1.07 (0.76-1.51)	67	1.92 (1.49-2.48)
C15 Oesophageal cancer	2017	208	1.39 (1.21-1.59)	317	1.09 (0.97-1.23)	689	1.00 (0.92-1.09)	135	0.94 (0.79-1.11)	173	1.49 (1.29-1.74)	178	2.18 (1.88-2.53)	317	3.51 (3.10-3.98)
C16 Stomach cancer	2720	304	1.18 (1.05-1.33)	614	1.13 (1.04-1.23)	868	1.00 (0.93-1.07)	288	1.10 (0.98-1.23)	239	1.08 (0.95-1.23)	221	1.43 (1.25-1.64)	186	1.20 (1.03-1.39)
C18 Colon cancer	1085	147	1.45 (1.23-1.71)	223	1.08 (0.94-1.24)	328	1.00 (0.89-1.12)	128	1.07 (0.90-1.27)	115	1.34 (1.12-1.62)	74	1.37 (1.09-1.73)	70	1.37 (1.07-1.74)
C19-C20 Rectal cancer	1131	142	1.29 (1.09-1.53)	225	1.07 (0.93-1.22)	335	1.00 (0.89-1.12)	126	1.00 (0.84-1.20)	135	1.39 (1.17-1.65)	78	1.26 (1.01-1.58)	90	1.41 (1.13-1.75)
C22 Liver cancer	2246	315	1.73 (1.55-1.94)	527	1.36 (1.24-1.50)	661	1.00 (0.92-1.08)	219	1.02 (0.89-1.17)	178	1.08 (0.93-1.26)	156	1.44 (1.23-1.69)	190	1.69 (1.45-1.96)
C32 Larynx cancer	208	26	1.83 (1.24-2.70)	27	0.91 (0.61-1.35)	42	1.00 (0.73-1.38)	22	1.35 (0.89-2.07)	27	2.05 (1.40-3.00)	23	2.83 (1.87-4.28)	41	4.95 (3.52-6.96)
C34 Lung cancer	4297	557	1.40 (1.29-1.52)	1102	1.38 (1.30-1.47)	1074	1.00 (0.94-1.06)	478	1.09 (1.00-1.20)	457	1.27 (1.16-1.39)	310	1.33 (1.18-1.48)	319	1.29 (1.15-1.45)
C80 Malignant neoplasm, without specification of site	1575	208	1.31 (1.14-1.51)	288	1.06 (0.93-1.19)	434	1.00 (0.91-1.10)	166	0.97 (0.83-1.14)	199	1.35 (1.17-1.55)	142	1.26 (1.07-1.49)	138	1.41 (1.18-1.68)
D38 Neoplasm of uncertain or unknown behaviour of middle ear and respiratory and intrathoracic organs	479	48	1.31 (0.98-1.75)	103	1.22 (0.99-1.50)	146	1.00 (0.84-1.19)	69	1.32 (1.04-1.68)	51	1.38 (1.04-1.82)	33	1.37 (0.97-1.94)	29	1.27 (0.87-1.85)
Any	19450	2287	1.30 (1.25-1.36)	4228	1.15 (1.11-1.18)	6028	1.00 (0.97-1.03)	2110	1.01 (0.97-1.06)	1880	1.16 (1.11-1.21)	1332	1.24 (1.18-1.31)	1585	1.45 (1.38-1.53)
**III Blood and immune-related**															
D64 Other anaemias	587	96	1.45 (1.18-1.78)	155	1.72 (1.45-2.04)	139	1.00 (0.84-1.19)	53	1.03 (0.78-1.36)	44	0.91 (0.67-1.22)	35	1.03 (0.74-1.44)	65	1.37 (1.06-1.78)
D69 Purpura and other haemorrhagic conditions	293	39	1.25 (0.90-1.72)	74	1.53 (1.20-1.96)	84	1.00 (0.80-1.25)	24	0.77 (0.52-1.17)	19	0.68 (0.43-1.08)	15	0.81 (0.49-1.35)	38	1.44 (1.03-2.04)
Any	1449	210	1.33 (1.15-1.52)	356	1.40 (1.25-1.57)	418	1.00 (0.90-1.11)	141	0.93 (0.79-1.10)	105	0.78 (0.64-0.95)	77	0.85 (0.68-1.06)	142	1.17 (0.99-1.40)
**IV Endocrine, nutritional and metabolic**															
E04 Other nontoxic goitre	351	32	0.88 (0.62-1.25)	58	0.84 (0.64-1.11)	149	1.00 (0.85-1.18)	50	0.76 (0.57-1.01)	27	0.57 (0.39-0.83)	22	0.70 (0.46-1.08)	13	0.53 (0.31-0.93)
E10-E14 Diabetes mellitus	12610	1713	1.54 (1.46-1.61)	2867	1.25 (1.20-1.3)	3957	1.00 (0.97-1.03)	1346	0.86 (0.81-0.91)	1099	0.91 (0.86-0.96)	818	1.01 (0.95-1.09)	810	1.02 (0.95-1.09)
E88 Other metabolic disorders	216	42	1.60 (1.17-2.18)	35	1.52 (1.08-2.14)	39	1.00 (0.72-1.38)	11	0.72 (0.40-1.30)	18	0.90 (0.56-1.43)	31	2.03 (1.43-2.90)	40	1.50 (1.09-2.07)
Any	14938	2029	1.48 (1.41-1.54)	3273	1.22 (1.17-1.26)	4697	1.00 (0.97-1.03)	1600	0.86 (0.82-0.91)	1320	0.90 (0.85-0.95)	988	1.00 (0.94-1.07)	1031	1.00 (0.94-1.06)
**V Psychiatric and behavioural**															
Less common psychiatric and behavioural ICD-10 codes combined†	1197	137	1.43 (1.21-1.70)	289	1.36 (1.20-1.54)	387	1.00 (0.90-1.11)	119	0.82 (0.69-0.99)	104	0.98 (0.81-1.20)	74	1.10 (0.87-1.38)	87	1.32 (1.06-1.65)
Any	1611	179	1.41 (1.21-1.63)	361	1.36 (1.22-1.53)	509	1.00 (0.91-1.10)	201	0.96 (0.84-1.11)	154	1.04 (0.88-1.22)	102	1.14 (0.94-1.39)	105	1.28 (1.05-1.56)
**VI Nerve-related**															

G40-G41 Epilepsy	487	64	1.92 (1.50-2.47)	122	1.66 (1.37-2.01)	144	1.00 (0.84-1.19)	52	1.07 (0.81-1.42)	34	0.97 (0.69-1.37)	32	1.49 (1.05-2.12)	39	1.80 (1.29-2.5)
G45 Transient cerebral ischaemic attacks and related syndromes	3758	402	1.25 (1.14-1.39)	563	1.10 (1.01-1.20)	1388	1.00 (0.95-1.06)	594	1.08 (0.99-1.17)	410	1.03 (0.93-1.13)	229	1.06 (0.93-1.21)	172	1.03 (0.89-1.21)
Any	7355	864	1.32 (1.24-1.42)	1442	1.15 (1.09-1.22)	2564	1.00 (0.96-1.04)	985	1.02 (0.96-1.09)	698	1.01 (0.94-1.09)	424	1.09 (0.99-1.2)	378	1.10 (0.99-1.22)
**VII Eye and adnexa**															
H25-H26 Cataract	6328	756	1.11 (1.03-1.19)	1533	1.01 (0.96-1.07)	2011	1.00 (0.95-1.05)	685	0.96 (0.89-1.03)	588	1.10 (1.02-1.20)	351	1.14 (1.02-1.27)	404	1.22 (1.10-1.35)
Any	9323	1083	1.09 (1.03-1.16)	2143	0.96 (0.92-1.00)	3120	1.00 (0.96-1.04)	1031	0.94 (0.88-1.00)	859	1.07 (1.00-1.14)	534	1.14 (1.05-1.24)	553	1.13 (1.04-1.24)
**VIII Ear and mastoid process**															
Any	2097	208	1.05 (0.91-1.20)	407	1.06 (0.95-1.17)	828	1.00 (0.93-1.08)	265	0.99 (0.88-1.12)	177	0.93 (0.80-1.07)	111	0.85 (0.70-1.03)	101	0.75 (0.62-0.92)
**IX Circulatory**															
I10 Essential (primary) hypertension	13950	1905	1.50 (1.43-1.57)	3176	1.21 (1.16-1.25)	4340	1.00 (0.97-1.03)	1660	1.06 (1.01-1.11)	1246	1.11 (1.05-1.17)	747	1.13 (1.05-1.22)	876	1.27 (1.18-1.36)
I11 Hypertensive heart disease	750	138	1.90 (1.60-2.26)	239	1.45 (1.27-1.67)	177	1.00 (0.85-1.17)	67	1.17 (0.92-1.49)	42	0.85 (0.63-1.15)	36	1.07 (0.77-1.48)	51	1.25 (0.94-1.67)
I25 Chronic ischaemic heart disease	17973	2424	1.44 (1.39-1.50)	4056	1.23 (1.19-1.27)	6084	1.00 (0.97-1.03)	2258	0.94 (0.90-0.98)	1567	0.96 (0.91-1.01)	852	0.98 (0.91-1.05)	732	1.02 (0.95-1.10)
I42 Cardiomyopathy	447	79	2.18 (1.73-2.74)	106	1.15 (0.94-1.41)	124	1.00 (0.83-1.21)	42	1.02 (0.75-1.38)	36	1.07 (0.77-1.49)	35	1.58 (1.13-2.22)	25	1.02 (0.68-1.53)
I51 Complications and ill-defined descriptions of heart disease	1083	164	1.69 (1.45-1.98)	236	1.32 (1.15-1.51)	350	1.00 (0.89-1.12)	99	0.85 (0.70-1.04)	97	1.07 (0.87-1.31)	74	1.23 (0.98-1.55)	63	1.13 (0.88-1.46)
I61 Intracerebral haemorrhage	6409	888	1.67 (1.56-1.78)	1891	1.37 (1.31-1.44)	1851	1.00 (0.95-1.05)	547	1.00 (0.92-1.09)	495	1.27 (1.16-1.39)	342	1.49 (1.33-1.65)	395	1.70 (1.53-1.88)
I63 Cerebral infarction	24260	3010	1.51 (1.45-1.56)	5479	1.25 (1.22-1.29)	8460	1.00 (0.98-1.02)	2929	0.99 (0.96-1.03)	2103	1.10 (1.06-1.15)	1232	1.19 (1.13-1.26)	1047	1.27 (1.20-1.36)
I64 Stroke, not specified as haemorrhage or infarction	1087	169	1.50 (1.29-1.75)	250	1.22 (1.07-1.40)	311	1.00 (0.89-1.12)	121	0.89 (0.74-1.07)	97	1.02 (0.83-1.24)	70	1.27 (1.00-1.62)	69	1.49 (1.17-1.91)
I65 Occlusion and stenosis of precerebral arteries	333	70	1.74 (1.37-2.22)	60	1.36 (1.04-1.77)	78	1.00 (0.79-1.26)	30	0.90 (0.62-1.29)	34	0.99 (0.70-1.38)	27	1.07 (0.73-1.57)	34	0.93 (0.66-1.32)
I66 Occlusion and stenosis of cerebral arteries	1235	174	1.59 (1.36-1.85)	360	1.32 (1.18-1.48)	377	1.00 (0.90-1.11)	127	0.87 (0.73-1.04)	89	0.90 (0.73-1.11)	54	1.21 (0.92-1.59)	54	1.60 (1.21-2.10)
I67 Other cerebrovascular diseases	12623	1526	1.26 (1.20-1.32)	2517	1.10 (1.05-1.14)	4553	1.00 (0.97-1.03)	1800	1.04 (0.99-1.09)	1135	1.00 (0.94-1.06)	612	1.03 (0.95-1.11)	480	0.97 (0.88-1.06)
I69 Sequelae of cerebrovascular disease	3892	662	1.93 (1.78-2.08)	951	1.33 (1.25-1.43)	1264	1.00 (0.94-1.06)	371	0.97 (0.87-1.07)	267	1.03 (0.91-1.17)	187	1.26 (1.09-1.46)	190	1.45 (1.25-1.68)
I80 Phlebitis and thrombophlebitis	348	41	1.46 (1.07-1.99)	55	0.91 (0.69-1.20)	118	1.00 (0.82-1.21)	53	1.32 (1.00-1.73)	34	1.31 (0.93-1.85)	19	1.24 (0.79-1.96)	28	2.17 (1.47-3.21)
Less common circulatory ICD-10 codes combined†	2448	354	1.54 (1.38-1.71)	540	1.16 (1.06-1.27)	785	1.00 (0.93-1.08)	252	0.91 (0.80-1.03)	222	1.06 (0.92-1.21)	148	1.10 (0.93-1.29)	147	1.00 (0.84-1.18)
Any	62339	7558	1.46 (1.43-1.49)	14525	1.23 (1.21-1.26)	21055	1.00 (0.99-1.01)	7319	0.99 (0.97-1.02)	5387	1.05 (1.03-1.08)	3302	1.13 (1.09-1.17)	3193	1.18 (1.13-1.22)
**X Respiratory**															
J12-J18 Pneumonia	14353	1880	1.24 (1.18-1.30)	3468	1.19 (1.14-1.23)	4407	1.00 (0.97-1.03)	1513	0.95 (0.90-1.00)	1270	1.01 (0.96-1.07)	831	1.02 (0.95-1.09)	984	1.06 (0.99-1.13)
J42 Unspecified chronic bronchitis	4097	687	1.50 (1.39-1.62)	1416	1.51 (1.43-1.59)	898	1.00 (0.93-1.07)	334	1.00 (0.90-1.12)	301	1.02 (0.91-1.14)	234	1.24 (1.09-1.41)	227	0.99 (0.87-1.13)
J44 Other chronic obstructive pulmonary disease	8497	1464	1.46 (1.38-1.53)	2746	1.51 (1.45-1.57)	1919	1.00 (0.95-1.05)	684	0.94 (0.87-1.01)	699	1.03 (0.96-1.11)	423	0.94 (0.85-1.04)	562	0.87 (0.80-0.95)
Any	30560	4076	1.31 (1.27-1.36)	7752	1.25 (1.22-1.28)	9097	1.00 (0.98-1.02)	3142	0.97 (0.93-1.00)	2707	1.03 (0.99-1.07)	1728	1.00 (0.96-1.05)	2058	1.00 (0.96-1.05)
**XI Digestive**															
K21 Gastro-oesophageal reflux disease	323	49	1.67 (1.25-2.22)	85	1.25 (1.00-1.58)	100	1.00 (0.81-1.23)	29	0.95 (0.65-1.37)	21	0.94 (0.61-1.45)	17	1.29 (0.80-2.09)	22	1.36 (0.88-2.13)

K25 Gastric ulcer	1236	147	1.28 (1.08-1.51)	256	1.12 (0.98-1.28)	370	1.00 (0.90-1.11)	139	1.10 (0.93-1.31)	127	1.21 (1.01-1.44)	85	1.21 (0.98-1.51)	112	1.32 (1.08-1.61)
K40 Inguinal hernia	3808	411	1.00 (0.91-1.11)	824	0.93 (0.86-1.00)	1327	1.00 (0.94-1.06)	435	0.98 (0.89-1.08)	355	1.01 (0.91-1.12)	218	0.89 (0.78-1.02)	238	0.86 (0.75-0.98)
K61 Abscess of anal and rectal regions	640	52	1.29 (0.98-1.71)	82	1.02 (0.81-1.28)	202	1.00 (0.86-1.16)	99	1.14 (0.93-1.39)	85	1.32 (1.06-1.63)	71	1.80 (1.42-2.28)	49	1.24 (0.92-1.66)
K70 Alcoholic liver disease	305	30	4.99 (3.46-7.19)	12	0.83 (0.47-1.48)	21	1.00 (0.64-1.55)	36	4.77 (3.42-6.66)	55	9.16 (7.03-11.94)	41	11.2 (8.24-15.21)	110	26.24 (21.28-32.34)
K74 Fibrosis and cirrhosis of liver	1212	165	2.14 (1.83-2.51)	290	1.74 (1.53-1.98)	340	1.00 (0.89-1.12)	95	0.82 (0.67-1.00)	116	1.32 (1.10-1.59)	82	1.47 (1.18-1.84)	124	2.26 (1.88-2.73)
K75 Other inflammatory liver diseases	410	55	1.92 (1.47-2.52)	65	1.26 (0.97-1.63)	133	1.00 (0.83-1.20)	59	1.22 (0.94-1.58)	45	1.36 (1.01-1.83)	28	1.29 (0.89-1.88)	25	1.39 (0.92-2.08)
K76 Other diseases of liver	770	112	1.54 (1.27-1.87)	119	1.23 (1.01-1.48)	208	1.00 (0.87-1.15)	74	0.83 (0.66-1.05)	91	1.15 (0.93-1.41)	48	0.88 (0.66-1.17)	118	1.58 (1.30-1.91)
K85-K86 Pancreatitis	968	112	1.34 (1.11-1.61)	218	1.21 (1.05-1.39)	298	1.00 (0.89-1.13)	117	1.07 (0.89-1.28)	77	0.86 (0.68-1.07)	53	0.88 (0.67-1.16)	93	1.36 (1.10-1.69)
K92 Other diseases of digestive system	2513	314	1.42 (1.27-1.59)	587	1.26 (1.16-1.38)	762	1.00 (0.93-1.08)	268	1.02 (0.90-1.15)	212	1.07 (0.93-1.22)	157	1.19 (1.02-1.40)	213	1.54 (1.34-1.77)
Any	30836	3374	1.22 (1.18-1.27)	6425	1.13 (1.10-1.16)	10343	1.00 (0.98-1.02)	3648	1.02 (0.99-1.05)	2838	1.01 (0.97-1.05)	1957	1.04 (0.99-1.08)	2251	1.08 (1.03-1.13)
**XII Skin and subcutaneous tissue**															
L08 Other local infections of skin and subcutaneous tissue	515	68	1.24 (0.98-1.59)	126	1.22 (1.01-1.47)	138	1.00 (0.84-1.19)	52	0.98 (0.75-1.30)	33	0.70 (0.49-0.98)	38	1.13 (0.82-1.56)	60	1.34 (1.02-1.75)
Any	2139	250	1.30 (1.15-1.48)	486	1.17 (1.07-1.29)	651	1.00 (0.92-1.08)	263	1.10 (0.97-1.24)	198	1.05 (0.91-1.21)	135	1.10 (0.92-1.30)	156	1.12 (0.95-1.32)
**XIII Musculoskeletal**															
M10 Gout	1010	149	1.84 (1.56-2.16)	209	1.16 (1.01-1.34)	250	1.00 (0.88-1.14)	111	1.25 (1.04-1.51)	109	1.82 (1.50-2.20)	79	2.22 (1.77-2.78)	103	2.73 (2.22-3.36)
M19 Other arthrosis	2006	228	1.18 (1.03-1.35)	440	1.00 (0.90-1.11)	653	1.00 (0.92-1.08)	233	1.09 (0.96-1.24)	191	1.19 (1.03-1.37)	120	1.18 (0.99-1.42)	141	1.18 (0.99-1.41)
M87 Osteonecrosis	304	26	1.46 (0.99-2.16)	48	1.47 (1.08-2.00)	75	1.00 (0.79-1.27)	27	1.01 (0.69-1.48)	39	1.91 (1.39-2.62)	37	2.85 (2.05-3.95)	52	3.77 (2.80-5.07)
Any	17404	1870	1.13 (1.08-1.18)	3769	1.00 (0.96-1.03)	6118	1.00 (0.97-1.03)	1936	0.99 (0.95-1.04)	1566	1.04 (0.99-1.09)	952	0.99 (0.93-1.06)	1193	1.04 (0.98-1.11)
**XIV Genitourinary**															
N40 Hyperplasia of prostate	3782	532	1.14 (1.05-1.25)	990	1.06 (0.99-1.13)	1260	1.00 (0.94-1.06)	442	0.99 (0.90-1.08)	258	0.76 (0.67-0.86)	147	0.69 (0.59-0.82)	153	0.63 (0.53-0.74)
Any	15105	1915	1.24 (1.18-1.30)	3578	1.10 (1.07-1.14)	5099	1.00 (0.97-1.03)	1641	0.93 (0.88-0.98)	1253	0.90 (0.85-0.95)	754	0.83 (0.77-0.89)	865	0.81 (0.75-0.86)
**XVIII Other symptoms, signs and abnormal findings**															
R53 Malaise and fatigue	208	34	1.45 (1.03-2.05)	40	0.91 (0.66-1.26)	52	1.00 (0.75-1.33)	14	0.69 (0.41-1.18)	22	1.16 (0.76-1.76)	18	1.03 (0.65-1.64)	28	1.80 (1.22-2.65)
R69 Unknown and unspecified causes of morbidity	8156	1354	1.38 (1.31-1.46)	1436	1.23 (1.16-1.29)	2047	1.00 (0.96-1.05)	914	1.00 (0.93-1.07)	854	0.97 (0.91-1.04)	733	1.05 (0.98-1.13)	818	1.09 (1.01-1.17)
R94 Abnormal results of function studies	340	39	1.71 (1.24-2.35)	68	1.78 (1.38-2.30)	72	1.00 (0.78-1.27)	41	1.50 (1.09-2.05)	37	1.58 (1.14-2.19)	28	1.62 (1.11-2.35)	55	2.53 (1.91-3.36)
R99 Other ill-defined and unspecified causes of mortality	380	47	1.41 (1.06-1.89)	105	1.54 (1.25-1.90)	120	1.00 (0.83-1.20)	37	0.77 (0.56-1.07)	30	1.13 (0.79-1.63)	21	1.49 (0.96-2.30)	20	1.85 (1.17-2.93)
Any	17617	2519	1.33 (1.28-1.39)	3631	1.17 (1.13-1.21)	5110	1.00 (0.97-1.03)	1960	0.96 (0.92-1.00)	1670	0.98 (0.93-1.02)	1300	1.04 (0.98-1.09)	1427	1.07 (1.02-1.13)
**XIX Injury, poisoning and other external causes**															
S22 Fracture of rib(s), sternum and thoracic spine	1023	105	1.07 (0.88-1.31)	211	0.92 (0.80-1.06)	274	1.00 (0.88-1.13)	93	1.01 (0.82-1.24)	95	1.07 (0.87-1.30)	96	1.40 (1.14-1.71)	149	1.56 (1.32-1.85)
S42 Fracture of shoulder and upper arm	688	64	1.21 (0.94-1.55)	142	1.16 (0.97-1.37)	199	1.00 (0.86-1.16)	75	1.18 (0.94-1.48)	68	1.25 (0.98-1.59)	50	1.19 (0.90-1.57)	90	1.60 (1.29-1.99)
S72 Fracture of femur	1077	139	1.38 (1.16-1.63)	285	1.19 (1.05-1.34)	294	1.00 (0.89-1.13)	115	1.10 (0.92-1.33)	92	1.21 (0.98-1.48)	56	1.11 (0.85-1.45)	96	1.77 (1.43-2.19)

Less common injury, poisoning and other external causes ICD-10 codes combined†	3137	297	1.13 (1.01-1.27)	705	1.07 (0.99-1.16)	1031	1.00 (0.94-1.07)	309	1.04 (0.93-1.17)	308	1.29 (1.16-1.45)	216	1.28 (1.12-1.47)	271	1.34 (1.18-1.52)
Any	11517	1146	1.14 (1.07-1.21)	2592	1.02 (0.98-1.07)	3784	1.00 (0.97-1.03)	1127	1.01 (0.95-1.07)	1049	1.15 (1.08-1.22)	775	1.18 (1.10-1.27)	1044	1.30 (1.22-1.39)
**XX External causes**															
V01-V99 Transport accidents	917	90	1.15 (0.93-1.42)	204	1.09 (0.94-1.26)	296	1.00 (0.88-1.13)	81	0.98 (0.78-1.22)	86	1.20 (0.97-1.49)	64	1.25 (0.97-1.60)	96	1.56 (1.26-1.92)
W00-W19 Falls	417	47	1.50 (1.12-2.01)	136	1.57 (1.30-1.89)	127	1.00 (0.83-1.21)	30	0.89 (0.62-1.27)	28	1.11 (0.76-1.61)	21	1.22 (0.79-1.88)	28	1.50 (1.02-2.21)
X60-X84 Intentional self-harm	274	28	1.47 (1.00-2.14)	87	1.55 (1.23-1.96)	76	1.00 (0.79-1.27)	23	1.20 (0.79-1.81)	18	1.19 (0.75-1.90)	14	1.49 (0.88-2.54)	28	2.61 (1.75-3.91)
Rest of V-Y	696	81	1.54 (1.23-1.92)	152	1.15 (0.97-1.36)	217	1.00 (0.86-1.16)	62	1.05 (0.82-1.35)	69	1.54 (1.21-1.95)	42	1.40 (1.03-1.90)	73	2.12 (1.66-2.71)
Any	2302	246	1.36 (1.20-1.54)	578	1.25 (1.14-1.36)	716	1.00 (0.92-1.08)	195	1.00 (0.87-1.16)	201	1.29 (1.12-1.48)	141	1.32 (1.12-1.56)	225	1.81 (1.57-2.08)

Cox models were stratified by age-at-risk and study area, and were adjusted for education and smoking.

† Included less common ICD-10 codes within the corresponding ICD-10 chapter which were not individually investigated in the present study. "Less common psychiatric and behavioural ICD-10 codes" consisted of ICD-10 codes F00-F99, excluding F32, F33, and F99. "Less common circulatory ICD-10 codes" consisted of ICD-10 codes I00-I99, excluding I10, I11, I20, I21, I24, I25, I27, I42, I46, I48-I51, I60-I67, I69, I70, I80, and I83. "Less common injury, poisoning and other external causes ICD-10 codes" consisted of ICD-10 codes S00-T98, excluding S06, S09, S22, S32, S42, S52, S62, S72, S82, S92, and T14.

Supplementary Table 7. Outcome classifications of pre-specified major diseases and aggregate endpoints

ICD-10 codes	Disease description
C00-C14, C15, C18-C20, C22, C32, C50 (women only)	Alcohol-related cancers (lips, oral cavity and pharynx, oesophagus, colon-rectum, liver, larynx, female breast)
E10-E14	Diabetes mellitus
I20-I25	Ischaemic heart disease
I60, I61, I63, I64	Stroke
K70, K74	Liver cirrhosis
V01-Y98	External causes
A15-A19, B90, C00-C14, C15, C18-C20, C22, C32, E10-E14, G40-G41, G45, I10, I11, I25, I42, I61, I63, I65, I66, I67, I69, J12-J18, K70, K74, K85-K86, V01-Y98	28 CKB WHO alcohol-related diseases[a]
C16, C34, C80, D38, D64, D69, E88, H25-H26, J42, J44, K21, K25, K61, K75, K76, K92, L08, M10, M19, M87, S00-T98 (excluding S06, S09, S32, S52, S62, S82, S92, T14), R53, R69, R94, R99, F00-F99 (excluding F32, F33, F99), I00-I99 (excluding I10, I11, I20, I21, I24, I25, I27, I42, I46, I48-I50, I60-I63, I65-I67, I69, I70, I83)	33 CKB new alcohol-associated diseases[b]
Any coded or uncoded disease events	All morbidity
All morbidity minus CKB WHO alcohol-related diseases, CKB new alcohol-associated diseases, and diseases negatively associated with alcohol in men or women	Non alcohol-related diseases

CKB, China Kadoorie Biobank; ICD-10, International Classification of Diseases, 10th Revision; WHO, World Health Organisation; FDR, false discovery rate.

[a] Diseases which were considered to be alcohol-related by the WHO and showed significant adverse associations at two-sided p<0.05 with alcohol drinking in the CKB were combined as “CKB WHO alcohol-related” diseases.

[b] Diseases showing significant adverse associations with alcohol drinking in men in CKB after multiple testing correction (FDR-adjusted p<0.05), but had not been classified as alcohol-related by WHO, were combined as “CKB new alcohol-associated diseases”.

Supplementary Figure 2. Adjusted HRs for ICD-10 Chapters I to VIII associated with usual alcohol intake, in male current drinkers

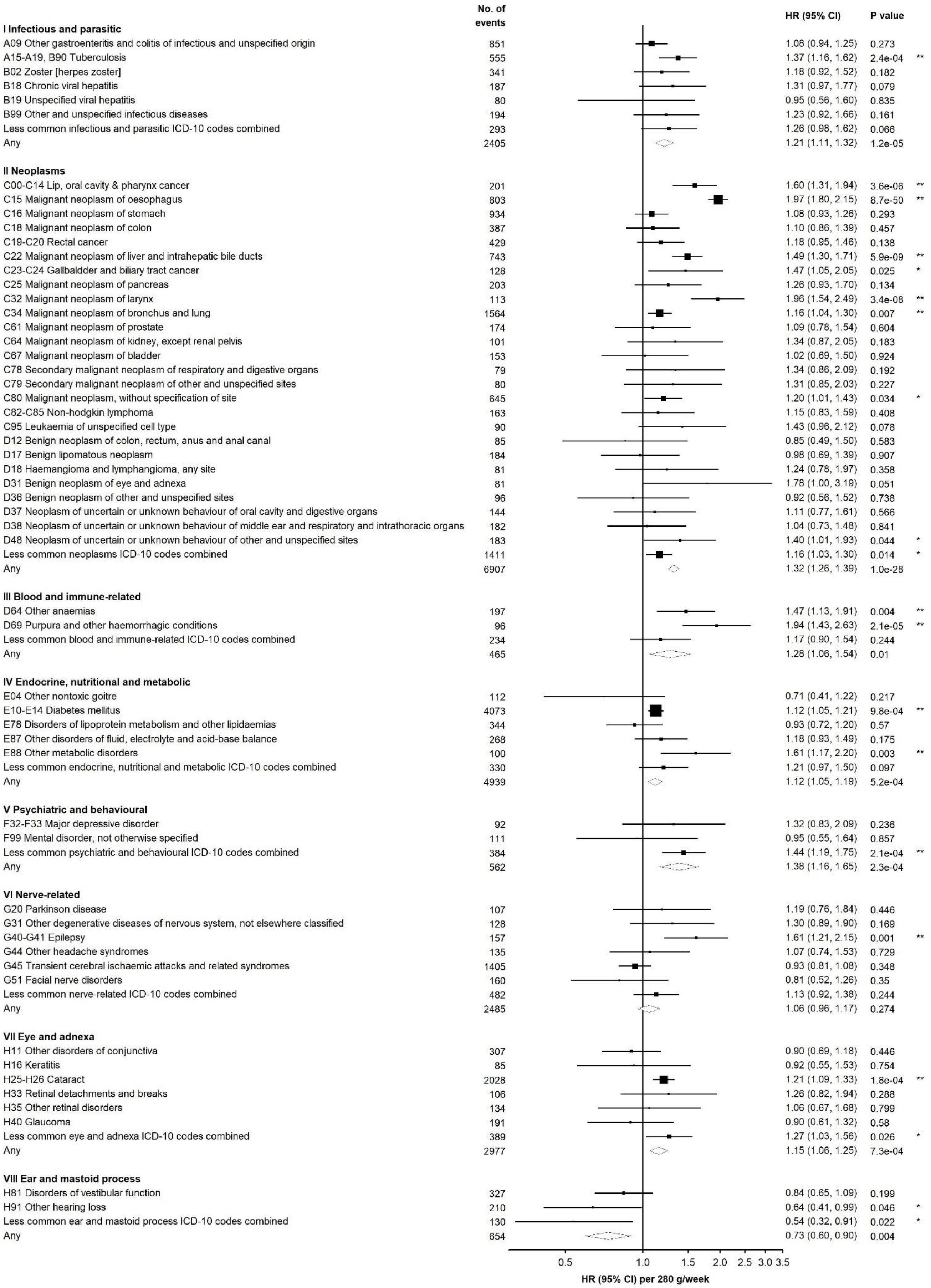

Cox models were stratified by age-at-risk and study area, and adjusted for education and smoking. Each solid square or diamond represents HR per 280 g/week higher usual alcohol intake among male current drinkers, with the area inversely proportional to the variance of the log HR. The horizontal lines indicate 95% CIs. All P values are two-sided. * indicate p<0.05; ** indicate FDR-adjusted p<0.05. CI, confidence interval; HR hazard ratio; ICD-10, International Classification of Diseases, 10th Revision.

Supplementary Figure 3. Adjusted HRs for ICD-10 Chapters IX to XI associated with usual alcohol intake, in male current drinkers

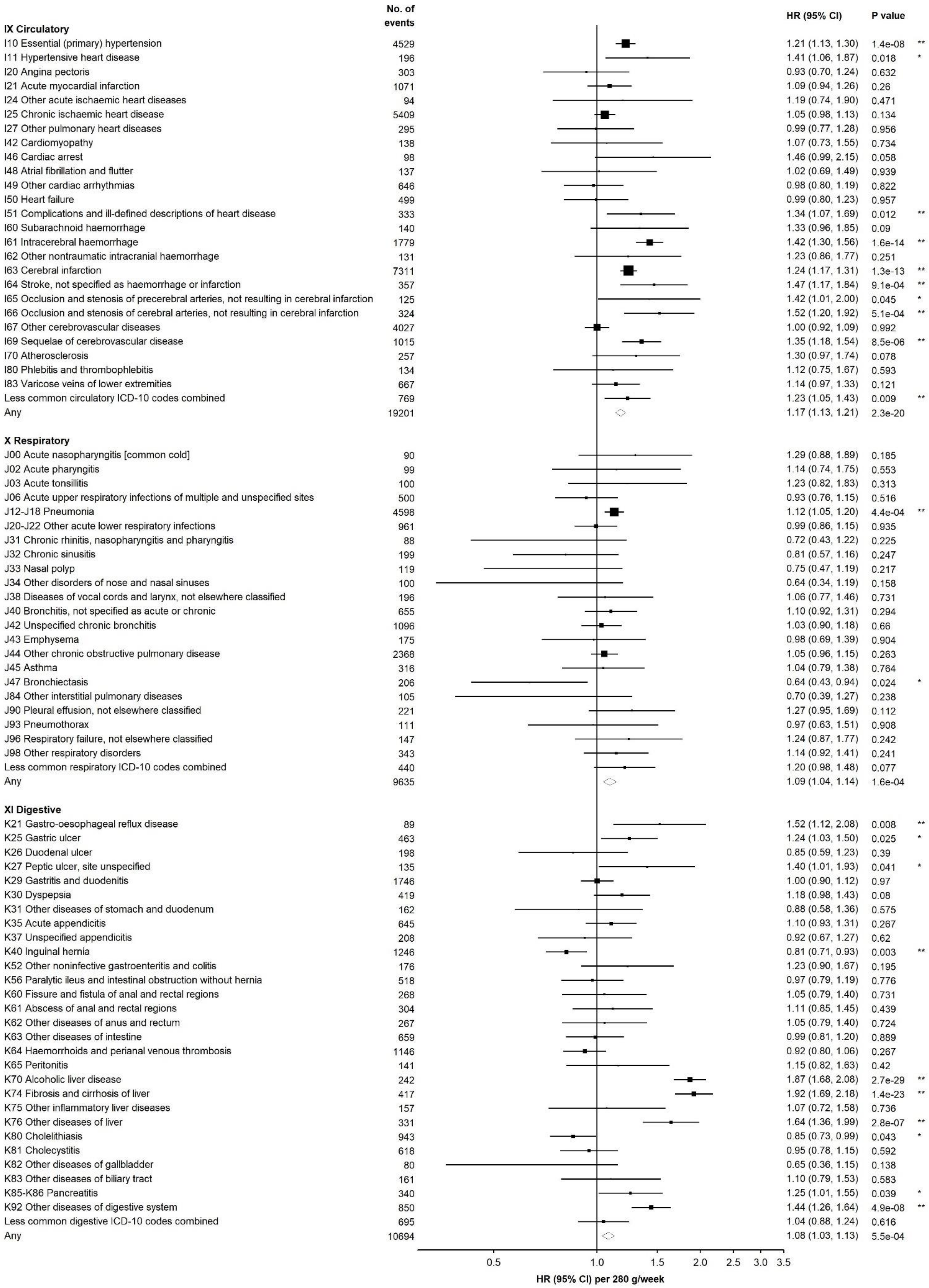

Cox models were stratified by age-at-risk and study area, and adjusted for education and smoking. Each solid square or diamond represents HR per 280 g/week higher usual alcohol intake among male current drinkers, with the area inversely proportional to the variance of the log HR. The horizontal lines indicate 95% CIs. All P values are two-sided. * indicate p<0.05; ** indicate FDR-adjusted p<0.05. CI, confidence interval; HR hazard ratio; ICD-10, International Classification of Diseases, 10th Revision.

Supplementary Figure 4. Adjusted HRs for ICD-10 Chapters XII to XX associated with usual alcohol intake, in male current drinkers

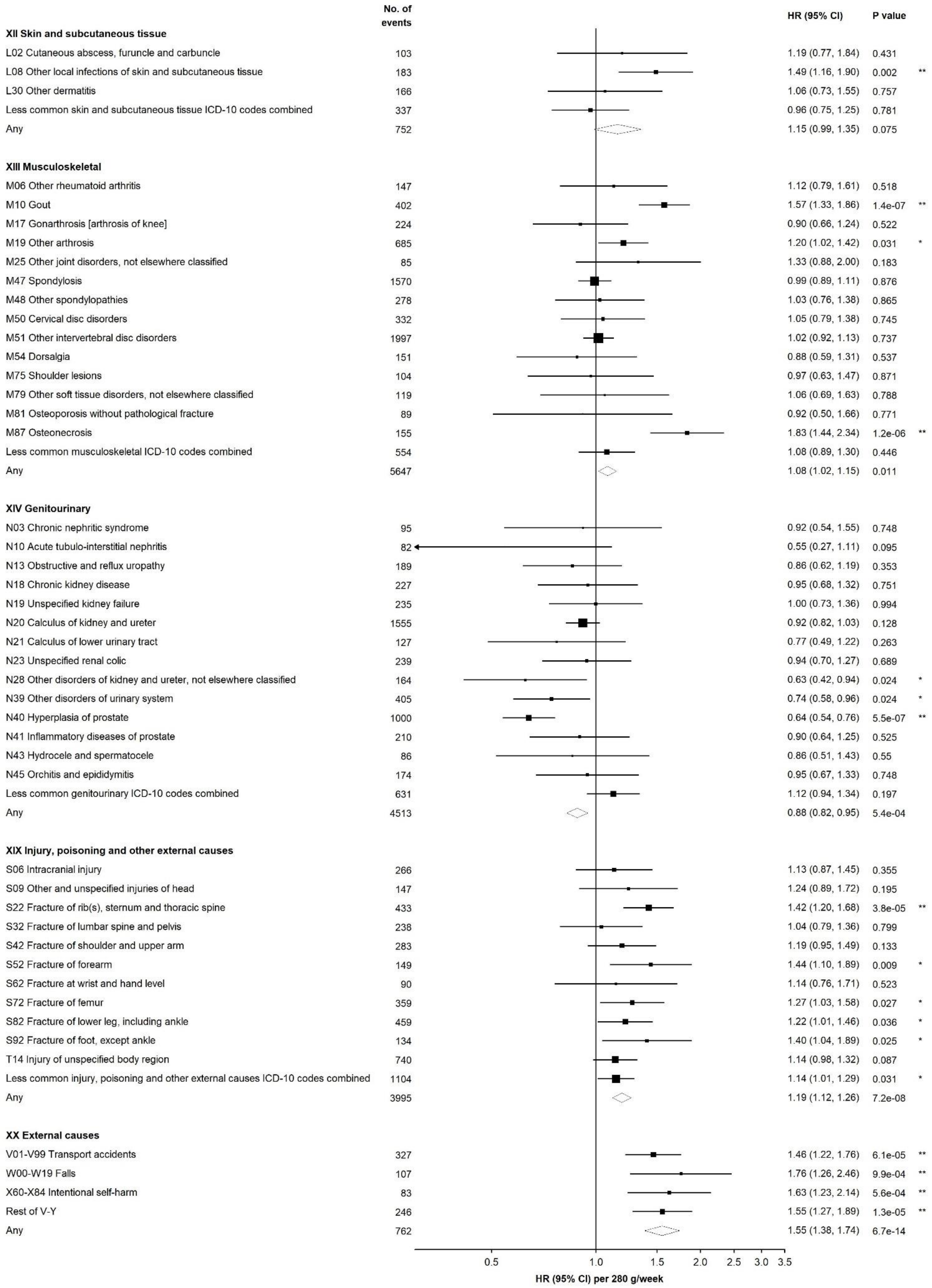

Cox models were stratified by age-at-risk and study area, and adjusted for education and smoking. Each solid square or diamond represents HR per 280 g/week higher usual alcohol intake among male current drinkers, with the area inversely proportional to the variance of the log HR. The horizontal lines indicate 95% CIs. All P values are two-sided. * indicate p<0.05; ** indicate FDR-adjusted p<0.05. CI, confidence interval; HR hazard ratio; ICD-10, International Classification of Diseases, 10th Revision.

Supplementary Figure 5. Adjusted HRs per 280 g/week higher usual alcohol intake for specific alcohol-associated diseases in male current drinkers, with further adjustments or exclusion of participants with baseline medical conditions

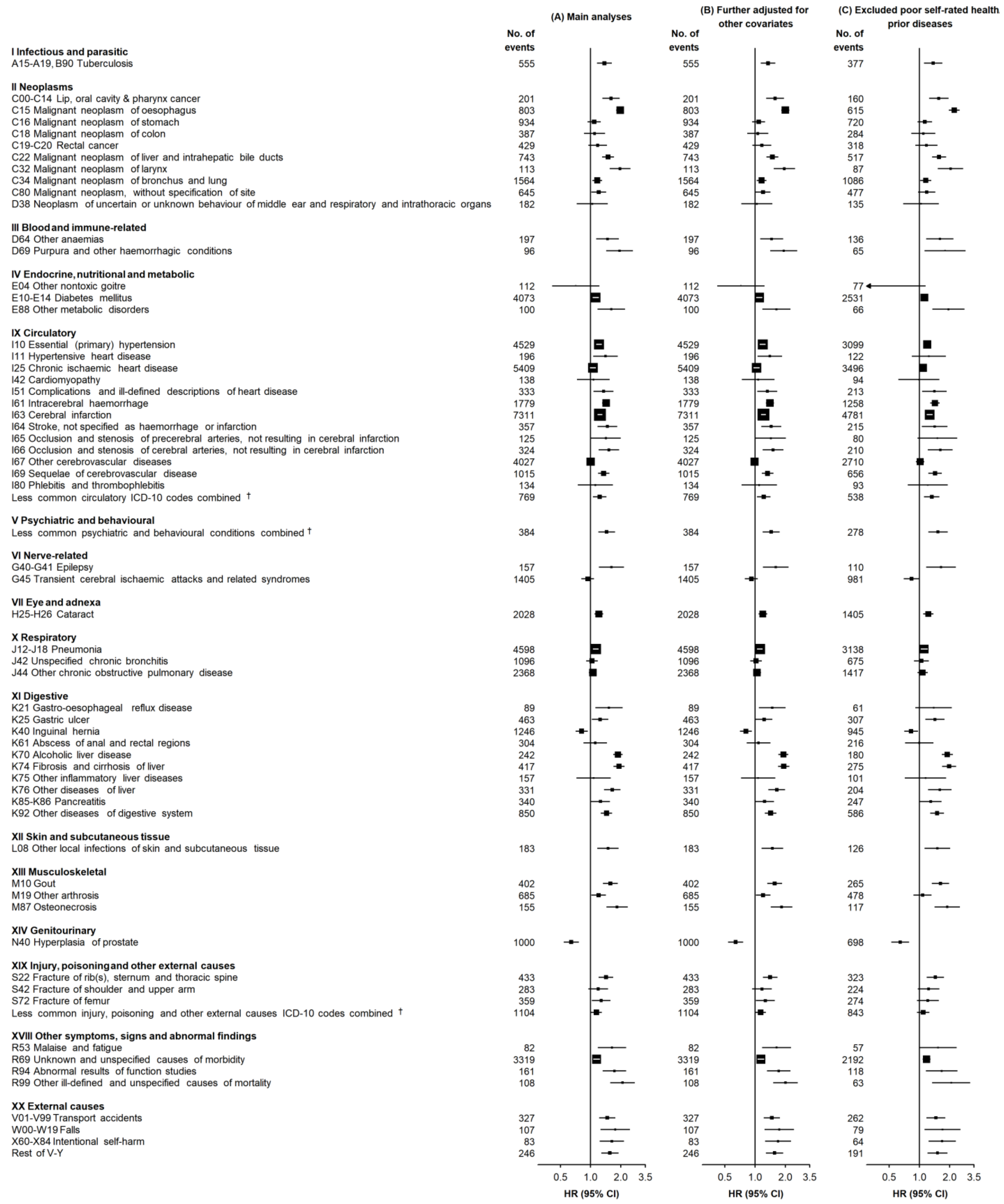

Cox models were stratified by age-at-risk and study area, and were adjusted for education and smoking in (A). (B) had the same model specification as in (A) plus further adjustments for income, physical activity, fruit intake, and body mass index. (C) had the same model specification as in (A) and excluded participants with poor self-rated health or prior chronic disease (i.e. self-reported history of coronary heart disease, stroke, transient ischaemic attack, diabetes, tuberculosis, emphysema/chronic bronchitis, liver cirrhosis/chronic hepatitis, peptic ulcer, gallstone/gallbladder disease, kidney disease, rheumatoid arthritis, and cancer) at baseline. Each solid square represents HR per 280 g/week higher usual alcohol intake among male current drinkers, with the area inversely proportional to the variance of the log HR. The horizontal lines indicate 95% CIs. † Included less common ICD-10 codes within the corresponding ICD-10 chapter which were not individually investigated in the present study. CI, confidence interval; HR hazard ratio; ICD-10, International Classification of Diseases, 10th Revision.

Supplementary Table 8. Adjusted HRs per 280 g/week higher usual alcohol intake for major diseases in male current drinkers, with further adjustments or exclusion of participants with baseline medical conditions

	Main analyses		Further adjusted for drinking duration		Further adjusted for other covariates (income, physical activity, fruit intake, BMI)		Excluded poor self-reported health / prior chronic diseases	
	**N**	**HR (95% CI)**	**N**	**HR (95% CI)**	**N**	**HR (95% CI)**	**N**	**HR (95% CI)**
Alcohol-related cancers	2452	1.63 (1.53-1.75)	2452	1.61 (1.50-1.73)	2452	1.63 (1.52-1.74)	1805	1.69 (1.56-1.83)
Diabetes mellitus	4073	1.12 (1.05-1.21)	4073	1.08 (1.01-1.16)	4073	1.10 (1.03-1.18)	2531	1.13 (1.03-1.23)
Ischaemic heart disease	6212	1.08 (1.01-1.15)	6212	1.07 (1.00-1.14)	6212	1.06 (1.00-1.14)	4054	1.11 (1.03-1.20)
Stroke	8731	1.28 (1.22-1.34)	8731	1.26 (1.19-1.32)	8731	1.26 (1.20-1.32)	5810	1.31 (1.24-1.39)
Liver cirrhosis	605	1.86 (1.70-2.03)	605	1.84 (1.68-2.01)	605	1.89 (1.72-2.07)	420	1.90 (1.71-2.11)
External causes	762	1.55 (1.38-1.74)	762	1.52 (1.35-1.71)	762	1.56 (1.39-1.75)	595	1.54 (1.35-1.76)
CKB new alcohol-associated diseases	15400	1.20 (1.16-1.24)	15400	1.19 (1.15-1.23)	15400	1.20 (1.16-1.24)	10750	1.22 (1.17-1.27)
CKB WHO alcohol-related diseases	25181	1.22 (1.19-1.25)	25181	1.20 (1.17-1.23)	25181	1.20 (1.17-1.24)	17631	1.24 (1.20-1.28)
All morbidity	44027	1.12 (1.10-1.14)	44027	1.11 (1.08-1.13)	44026	1.11 (1.09-1.14)	32508	1.13 (1.10-1.15)

CKB, China Kadoorie Biobank; WHO, World Health Organisation; HR, hazard ratio; CI, confidence interval; BMI, body mass index.
Cox models were stratified by age-at-risk and study area, and were adjusted for education and smoking in the main analyses. Prior chronic diseases included self-reported history of coronary heart disease, stroke, transient ischaemic attack, diabetes, tuberculosis, emphysema/chronic bronchitis, liver cirrhosis/chronic hepatitis, peptic ulcer, gallstone/gallbladder disease, kidney disease, rheumatoid arthritis, and cancer.

Supplementary Figure 6. Adjusted HRs for CKB WHO alcohol-related diseases, CKB new alcohol-associated diseases and all morbidity per 280 g/week higher usual alcohol intake, by population subgroups in male current drinkers

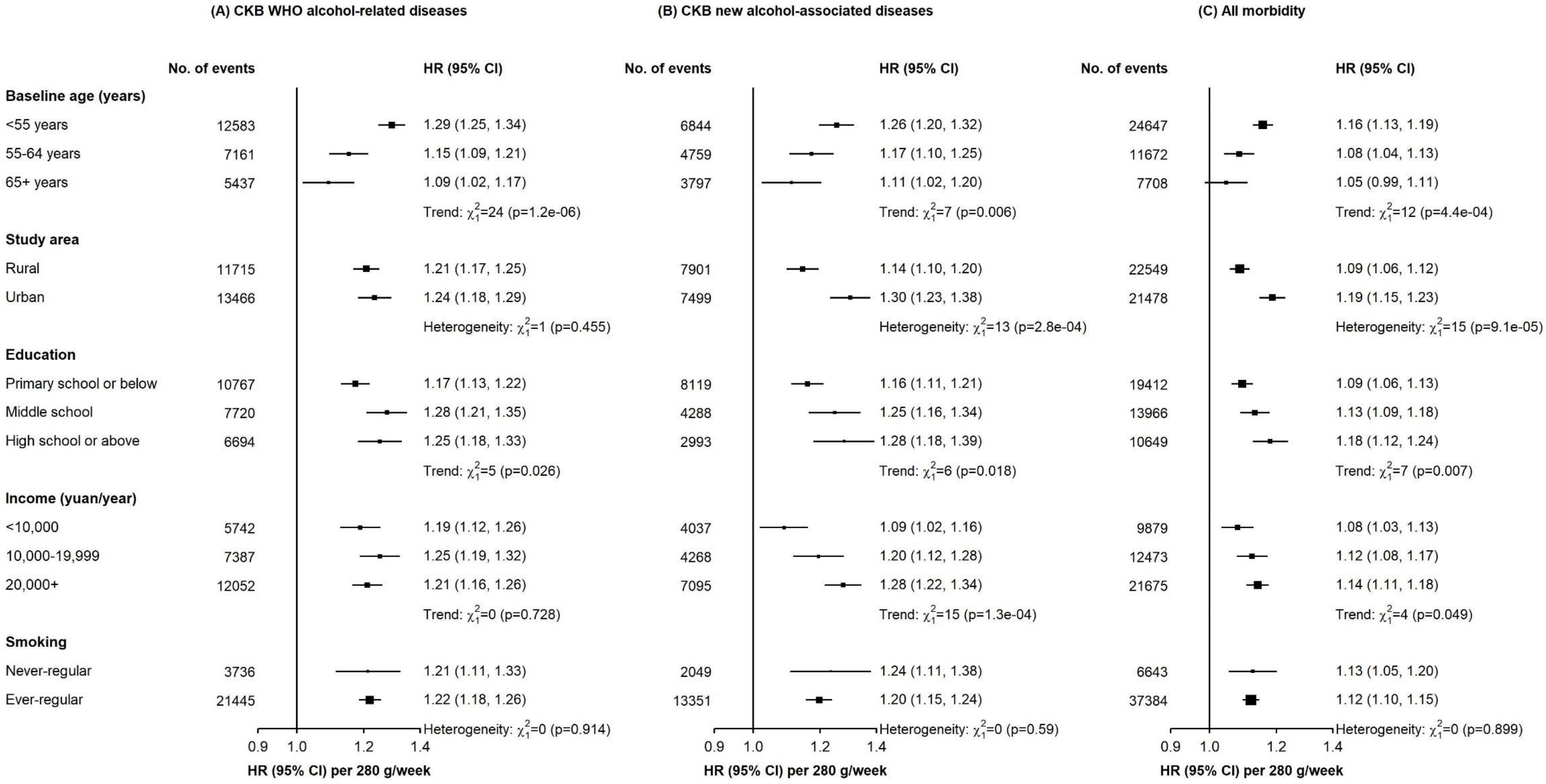

Cox models were stratified by age-at-risk and study area, and adjusted for education and smoking. Each solid square represents HR per 280 g/week higher usual alcohol intake among male current drinkers, with the area inversely proportional to the variance of the log HR. The horizontal lines indicate 95% CIs. All P values are two-sided. CI, confidence interval; HR hazard ratio; ICD-10, International Classification of Diseases, 10th Revision.

Supplementary Figure 7. Associations of aggregate disease categories with self-reported alcohol intake and with genotype-predicted mean male alcohol intake categories, in women

Conventional epidemiological analyses

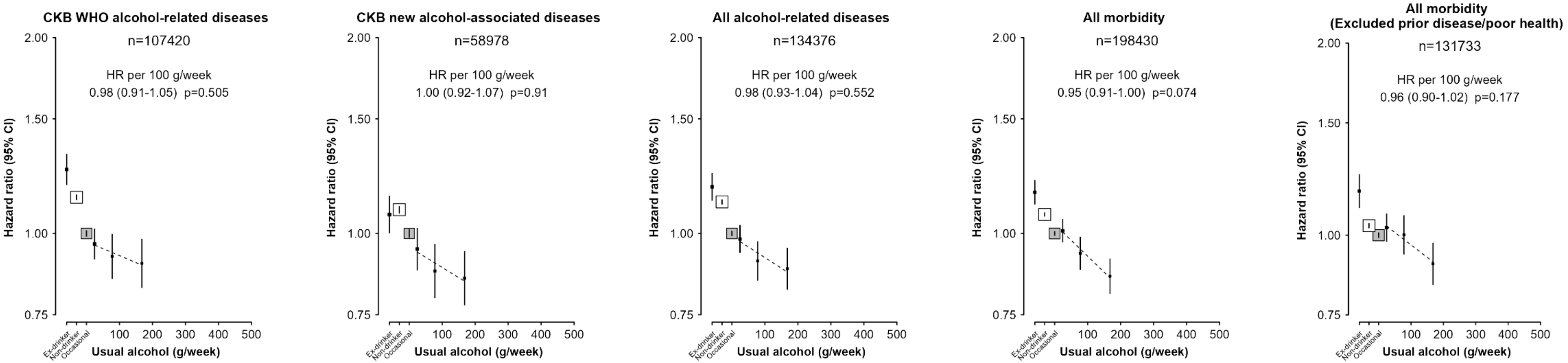

Genetic epidemiological analyses

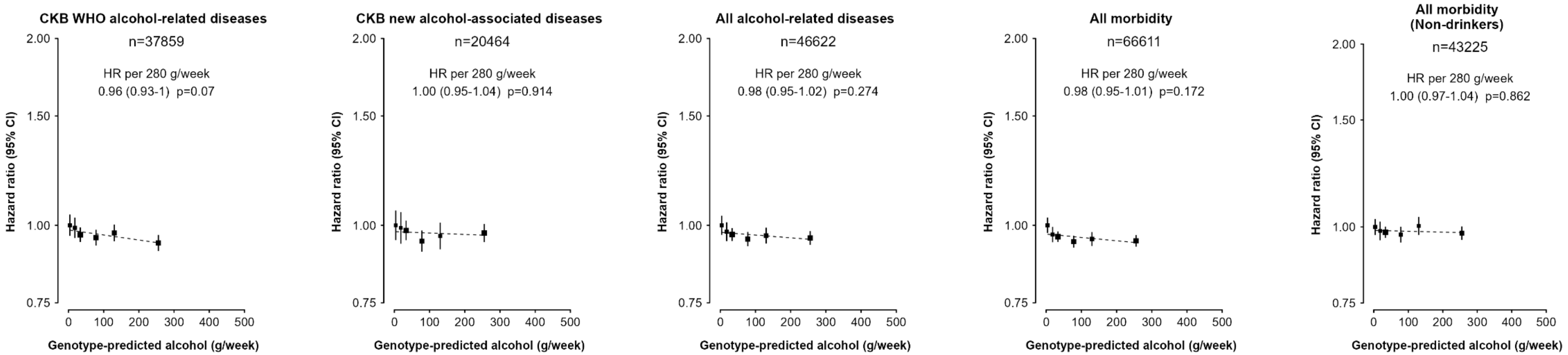

Conventional epidemiological analyses relate self-reported drinking patterns to risks of diseases (reference group = occasional drinkers), using Cox models stratified by age-at-risk and study area and adjusted for education and smoking. Within current drinkers, HRs were plotted against usual alcohol intake and were calculated per 100 g/week higher usual alcohol intake. Genetic epidemiological analyses relate genetic categories to risks of diseases (reference group = genotype group with lowest mean genotype-predicted mean male alcohol intake), using Cox models by age-at-risk and study area and adjusted for 11 genomic principal components. The HR per 280 g/week higher genotype-predicted mean male alcohol intake is calculated from the inverse-variance-weighted mean of the slopes of the fitted lines in each study area. Each box represents HR with the area inversely proportional to the variance of the group-specific log hazard within subplot. The vertical lines indicate group-specific 95% CIs. All P values are two-sided. CI, confidence interval; HR hazard ratio; CKB, China Kadoorie Biobank; ICD-10, International Classification of Diseases, 10th Revision; WHO, World Health Organisation.

Supplementary Table 9. Genotype distribution and allele frequencies of *ALDH2*-rs671 and *ADH1B*-rs1229984 across the ten study areas

	No of men and women genotyped	*ALDH2*-rs671				*ADH1B*-rs1229984			
		GG	AG	AA	A-allele frequency[a,b]	GG	AG	AA	A-allele frequency[a,b]
**Study area[c]**									
Harbin (Urban)	17879	12520	4876	483	0.16	1928	7976	7975	0.67
Qingdao (Urban)	11944	7957	3615	372	0.18	1190	5123	5631	0.69
Suzhou (Urban)	15189	9035	5389	765	0.23	1306	6226	7657	0.71
Liuzhou (Urban)	14018	8048	5118	852	0.24	1237	5840	6941	0.70
Haikou (Urban)	7693	3860	3160	673	0.29	538	2935	4220	0.74
Gansu (Rural)	16098	11833	3939	326	0.14	2083	7518	6497	0.64
Henan (Rural)	17762	13392	4073	297	0.13	2022	7982	7758	0.66
Sichuan (Rural)	16403	10725	5120	558	0.19	1687	7078	7638	0.68
Zhejiang (Rural)	18022	9378	7198	1446	0.28	1442	7336	9244	0.72
Hunan (Rural)	16339	8766	6453	1120	0.27	1270	6410	8659	0.73
**All areas**	**151347**	**95514**	**48941**	**6892**	**0.21**	**14703**	**64424**	**72220**	**0.69**

[a] A-alleles decrease alcohol tolerability. Genotype distributions did not deviate from Hardy-Weinberg equilibrium within study areas.
[b] Corresponding frequencies in European-origin populations (1KGP) are 0.00 (*ALDH2*-rs671) and 0.03 (*ADH1B*-rs1229984).
[c] Within rural and urban level, the study areas are ordered from North to South.

Supplementary Table 10. Baseline characteristics and alcohol drinking patterns by *ALDH2*-rs671 genotype, and by *ADH1B*-rs1229984 genotype

			ALDH2-rs671				*ADH1B*-rs1229984			
	Overall in CKB study	Genotyped subset	AA	AG	GG	P value for trend / G-allele	AA	AG	GG	P value for trend / G-allele
**Men**										
N	210205	61046	2770	19905	38371		29187	26104	5755	
Mean age, years	52.8	52.9	53	52.9	52.9	0.11	52.9	52.9	52.6	0.13
Education > 6 years, %	57.8	57.8	56.6	57.5	57.9	0.50	57.7	57.9	57.1	0.44
Household income > 20,000 yuan/year, %	45.6	44.7	44.5	44.9	44.7	0.37	45	44.4	44.6	0.16
Current smokers, %	61.1	60.9	59.8	61	60.9	0.33	61	60.7	61	0.53
Infrequent fresh fruit intake, %	77.0	76.5	73.5	74.2	77.9	2.6×10^{-18}	76.3	76.7	76.9	0.90
Physical activity, mean MET-h/d	22.0	22.1	21.4	21.9	22.1	0.14	22	22.2	21.8	0.93
Mean alcohol intake overall, g/week[a]	106.1	108.4	2.4	37.4	162.4	$<1 \times 10^{-100}$	100.9	109	162.3	5.5×10^{-41}
Ever-regular drinking, %	42.0	42.5	2.4	22.3	56.9	$<1 \times 10^{-100}$	41.1	42.1	52.4	8.7×10^{-32}
**Women**										
N	302519	90301	4122	29036	57143		43033	38320	8948	
Mean age, years	51.5	51.5	51.9	51.5	51.4	0.17	51.4	51.5	51.4	0.30
Education > 6 years, %	43.3	43.3	43.7	43.4	43.1	0.04	43.6	43.1	42.4	9.7×10^{-4}
Household income > 20,000 yuan/year, %	40.7	39.5	39.3	39.4	39.7	0.70	39.7	39.4	38.9	0.17
Current smokers, %	2.4	2.3	2.7	2.2	2.4	0.03	2.3	2.3	2.6	0.85
Infrequent fresh fruit intake, %	68.2	67.5	65.9	67.4	67.6	0.11	67.4	67.6	68	0.29
Physical activity, mean MET-h/d	20.4	20.5	20.5	20.5	20.5	0.58	20.4	20.5	20.3	0.97
Mean alcohol intake overall, g/week[a]	4.1	4.1	0.7	1.9	5.4	8.2×10^{-47}	3.7	4.1	6	1.8×10^{-6}
Ever-regular drinking, %	2.9	2.9	0.3	1.2	4	4.2×10^{-40}	2.7	3	4.1	1.3×10^{-6}

MET-h/d, metabolic equivalent of task per hour per day.
Prevalences and means are adjusted for study area and (where appropriate) age (in 10-year intervals) structure of the CKB randomly selected genotyped subset, using direct standardisation separately by sex.
The P value for trend is from an inverse-variance-weighted meta-analysis across ten areas, with within-area per G-allele effect adjusted (where appropriate) for age and genomic principal components using multiple linear regression models. All P values are two-sided.
[a] Calculations assign an intake of 5 g/week to occasional drinkers, and exclude ex-drinkers.

Supplementary Table 11. Baseline characteristics and alcohol drinking patterns by six genetic categories (C1-C6)

	Genotyped subset	Genetic category						P value for trend by mean male intake
		C1	C2	C3	C4	C5	C6	
**Men**								
N	61046	4269	6356	11984	13542	9057	15838	
Mean age, years	52.9	53.1	52.9	53.1	53.0	52.6	52.9	0.03
Education > 6 years, %	57.8	56.0	56.8	58.9	57.0	58.3	57.8	0.85
Household income > 20,000 yuan/year, %	44.7	43.6	45.8	44.7	45.1	43.7	44.5	0.85
Current smokers, %	60.9	59.6	60.7	61.6	60.7	61.1	60.8	0.94
Infrequent fresh fruit intake, %	76.5	73.5	73.3	75.3	75.5	78.5	79.6	6.3×10^{-16}
Physical activity, mean MET-h/d	22.1	21.9	21.8	22.0	22.1	22.2	22.2	0.31
Mean alcohol intake overall, g/week[a,b]	108.4	4.0	18.3	33.5	78.2	130.2	255.4	$<1 \times 10^{-100}$
Ever-regular drinking, %[a]	42.5	3.0	15.1	20.4	39.1	59.9	74.0	$<1 \times 10^{-100}$
**Women**								
N	90301	6454	9740	17191	20015	13075	23826	
Mean age, years	51.5	51.7	51.5	51.5	51.4	51.5	51.4	0.68
Education > 6 years, %	43.3	44.1	43.7	43.2	43.7	42.6	42.8	0.04
Household income > 20,000 yuan/year, %	39.5	38.8	39.2	39.4	39.8	39.1	39.7	0.32
Current smokers, %	2.3	2.4	2.1	2.4	2.1	2.3	2.7	0.08
Infrequent fresh fruit intake, %	67.5	66.3	68.1	67.3	67.5	67.3	68.2	0.09
Physical activity, mean MET-h/d	20.5	20.4	20.6	20.4	20.5	20.3	20.6	0.27
Mean alcohol intake overall, g/week[a,b]	4.1	0.6	1.9	1.2	3.5	5.4	7.8	1.1×10^{-57}
Ever-regular drinking, %[a]	2.9	0.2	0.8	0.8	2.1	4.5	5.8	1.2×10^{-77}

MET-h/d, metabolic equivalent of task per hour per day.
Prevalences or means (except for those for alcohol drinking) are adjusted for area, genomic principal components and (where appropriate) age, using multiple linear regression models.
The P value for trend is from an inverse-variance-weighted meta-analysis across ten areas, with within-area slopes adjusted (where appropriate) for age and genomic principal components using multiple linear regression models. All P values are two-sided.
[a] Prevalences or means of alcohol consumption are unadjusted.
[b] Calculations assign an intake of 5 g/week to occasional drinkers, and exclude ex-drinkers.

Supplementary Table 12. Adjusted HRs per 280 g/week higher genotype-predicted alcohol intake with alcohol-related diseases and overall morbidity, sensitivity analyses in men

	Main approach<br>HR (95% CI)	Main approach (area-adjusted)[a]<br>HR (95% CI)	Area-stratified 2SLS (additive) approach[b]<br>HR (95% CI)	Area-adjusted 2SLS (additive) approach[c]<br>HR (95% CI)	Area-stratified 2SLS (genotype) approach[d]<br>HR (95% CI)	Area-adjusted 2SLS (genotype) approach[e]<br>HR (95% CI)
CKB WHO alcohol-related diseases	1.14 (1.09-1.20)	1.15 (1.10-1.21)	1.12 (1.07-1.18)	1.16 (1.10-1.22)	1.14 (1.09-1.20)	1.17 (1.11-1.23)
CKB new alcohol-associated diseases	1.06 (1.01-1.12)	1.10 (1.03-1.17)	1.07 (1.02-1.13)	1.12 (1.04-1.20)	1.07 (1.01-1.12)	1.11 (1.04-1.18)
All alcohol-related diseases	1.11 (1.07-1.16)	1.13 (1.08-1.18)	1.10 (1.05-1.15)	1.14 (1.09-1.20)	1.11 (1.07-1.15)	1.15 (1.09-1.20)
All morbidity	1.07 (1.03-1.11)	1.09 (1.05-1.13)	1.07 (1.03-1.10)	1.10 (1.05-1.14)	1.07 (1.03-1.11)	1.10 (1.05-1.14)

2SLS, two-stage least-squares; HR, hazard ratio; CI, confidence interval; PC, principal components.

[a] Estimated using the same approach as the main approach but with an area-adjusted model, i.e. using Cox regression model stratified by age-at-risk and ten study areas and adjusted for 11 genomic PCs.

[b] Estimated using 2SLS approach within each study area and meta-analysed with inverse-variance-weighted method across ten study areas. In the first stage, total alcohol intake (assigning 5 g/week to occasional drinkers and excluding ex-drinkers) was regressed against rs671 (continuous, per G allele), rs1229984 (continuous, per G allele) and covariates (age, genomic PCs) using linear regression in the population subset of genotyped men within each study area. In the second stage, genetically-predicted alcohol intake (assigned to all genotyped participants in the corresponding study area as a result of the first stage) was related to disease risk using Cox regression stratified by age-at-risk and adjusted for genomic PCs to yield area-specific effect estimates, which were then meta-analysed with inverse-variance-weighted method.

[c] Estimated using 2SLS approach with an area-adjusted model. In the first stage, total alcohol intake (assigning 5 g/week to occasional drinkers and excluding ex-drinkers) was regressed against rs671 (continuous, per G allele), rs1229984 (continuous, per G allele) and covariates (age, ten study areas, 11 genomic PCs) using linear regression in the population subset of genotyped men. In the second stage, genetically-predicted alcohol intake (assigned to all genotyped participants as a result of the first stage) was related to disease risk using Cox regression stratified by age-at-risk and ten study areas and adjusted for 11 genomic PCs.

[d] Estimated using 2SLS approach as described above in (b), except that rs671 and rs1229984 were modelled as categorical variables (each AA, AG, GG) in the first stage.

[e] Estimated using 2SLS approach as described above in (c), except that rs671 and rs1229984 were modelled as categorical variables (each AA, AG, GG) in the first stage.

Supplementary Figure 8. Total expected hospitalisations overall and by major disease categories in ever- and never-regular drinkers from age-at-risk of 35 years among men

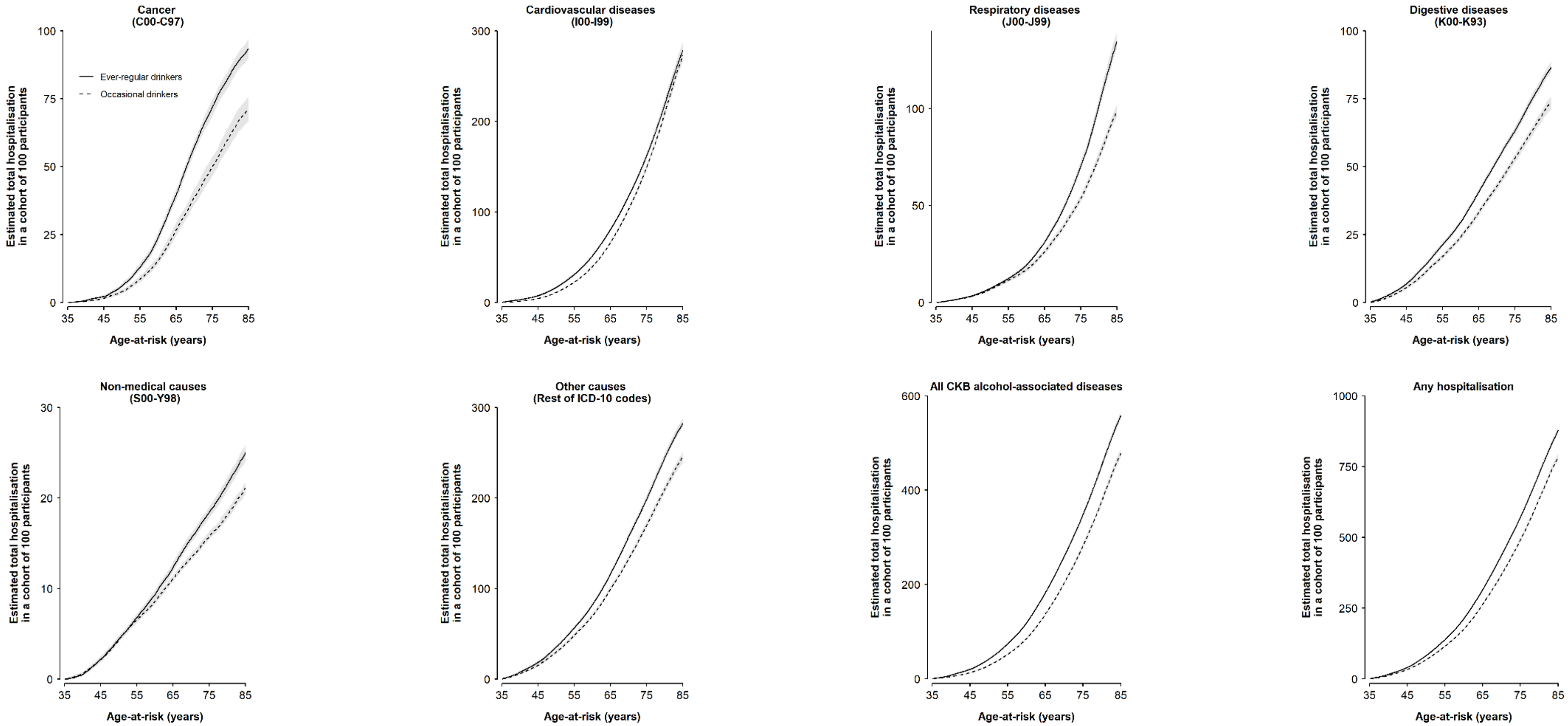

The solid and dashed lines indicate the estimated total hospitalisation of specific causes in a cohort of 100 participants in male ever-regular drinkers and male occasional drinkers, respectively. Grey bands show 95% confidence intervals. CKB, China Kadoorie Biobank; ICD-10, International Classification of Diseases, 10th Revision. “All CKB alcohol-related diseases” include disease events from “CKB WHO alcohol-related diseases” or “CKB new alcohol-associated diseases”.

Supplementary Table 13. ICD-10 code mapping of CKB significant WHO alcohol-related diseases to alcohol-related diseases based on WHO-classifications

WHO alcohol-related diseases	WHO codes	CKB codes*
Tuberculosis	A15−A19, B90	A15−A19, B90
HIV/AIDS†	B20−B24	
Lower respiratory infections	J09−J22, P23, U04	J12-J18
Lip, oral cavity and pharynx cancer	C00-C14	C00-C14
Oesophageal cancer	C15	C15
Colorectal cancer	C18-C21	C18; C19-C20
Liver cancer	C22	C22
Larynx cancer	C32	C32
Female breast cancer†	C50	
Diabetes mellitus	E10–E14 (minus E10.2–E10.29, E11.2–E11.29, E12.2, E13.2–E13.29, E14.2)	E10-E14
Alcohol use disorders†	F10, G72.1, Q86.0, X45	
Epilepsy	G40–G41	G40–G41
Hypertensive heart disease	I10–I15	I10; I11
Ischaemic heart disease	I20–I25	I25 (also significant when analysed as pre-specified major disease [I20-I25])
Ischaemic stroke	G45–G46.8, I63–I63.9, I65–I66.9, I67.2–I67.848, I69.3– I69.4	G45; I63; I65; I66; I67; I69
Haemorrhagic stroke	I60–I62.9, I67.0–I67.1, I69.0–I69.298	I61; I67; I69
Cardiomyopathy, myocarditis, endocarditis	I30–I33, I38, I40, I42	I42
Cirrhosis of the liver	K70, K74	K70; K74
Pancreatitis	K85–K86	K85–K86
Road injury	V01–V04, V06, V09–V80, V87, V89, V99*	V01-V99
Poisonings	X40, X43, X46–X48, X49	Rest of V-Y
Falls	W00–W19	W00-W19
Fire, heat and hot substances	X00–X19	Rest of V-Y
Drowning	W65–W74	Rest of V-Y
Exposure to mechanical forces	W20–W38, W40–W43, W45, W46, W49–W52, W75, W76	Rest of V-Y
Other unintentional injuries	Rest of V, W39, W44, W53–W64, W77–W99, X20–X29, X50– X59, Y40–Y86, Y88, Y89	Rest of V-Y
Self-harm	X60–X84, Y870	X60-X84
Interpersonal violence	X85–Y09, Y871	Rest of V-Y

CKB, China Kadoorie Biobank; ICD-10, International Classification of Diseases, 10th Revision; WHO, World Health Organisation.
* CKB codes refer to ICD-10 codes that were significantly associated with alcohol intake in the present study.
† Recorded numbers of cases for these diseases were too low in the present study for these individual diseases to be investigated.
