# Supplementary material for: Alcohol consumption and risks of more than 200 diseases in Chinese men
Source: Nat Med. 2023 Jun 8;29(6):1476–86. doi: 10.1038/s41591-023-02383-8 (PMC10287564; doi:10.1038/s41591-023-02383-8)
Supplement: Supplementary file 2 — Reporting Summary [file 41591_2023_2383_MOESM2_ESM.pdf]

nature portfolio

Corresponding author(s): Iona Y Millwood, Zhengming Chen

Last updated by author(s): Apr 28, 2023

Reporting Summary

Nature Portfolio wishes to improve the reproducibility of the work that we publish. This form provides structure for consistency and transparency in reporting. For further information on Nature Portfolio policies, see our Editorial Policies and the Editorial Policy Checklist.

Statistics

For all statistical analyses, confirm that the following items are present in the figure legend, table legend, main text, or Methods section.

n/a	Confirmed	
☐	☒	The exact sample size (*n*) for each experimental group/condition, given as a discrete number and unit of measurement
☐	☒	A statement on whether measurements were taken from distinct samples or whether the same sample was measured repeatedly
☐	☒	The statistical test(s) used AND whether they are one- or two-sided<br>*Only common tests should be described solely by name; describe more complex techniques in the Methods section.*
☐	☒	A description of all covariates tested
☐	☒	A description of any assumptions or corrections, such as tests of normality and adjustment for multiple comparisons
☐	☒	A full description of the statistical parameters including central tendency (e.g. means) or other basic estimates (e.g. regression coefficient) AND variation (e.g. standard deviation) or associated estimates of uncertainty (e.g. confidence intervals)
☐	☒	For null hypothesis testing, the test statistic (e.g. *F*, *t*, *r*) with confidence intervals, effect sizes, degrees of freedom and *P* value noted<br>*Give P values as exact values whenever suitable.*
☒	☐	For Bayesian analysis, information on the choice of priors and Markov chain Monte Carlo settings
☒	☐	For hierarchical and complex designs, identification of the appropriate level for tests and full reporting of outcomes
☐	☒	Estimates of effect sizes (e.g. Cohen's *d*, Pearson's *r*), indicating how they were calculated

Our web collection on statistics for biologists contains articles on many of the points above.

Software and code

Policy information about availability of computer code

Data collection	A range of bespoke CKB IT systems was used for data collection (see https://www.ckbiobank.org/study-resources/management-systems)
Data analysis	All analyses were performed using R software (version 4.0.5). The codes used for the data analyses in our study can be made available by contacting the corresponding authors. Access to code will be granted for requests for academic use within 4 weeks of application. Code will be released by the corresponding authors.

For manuscripts utilizing custom algorithms or software that are central to the research but not yet described in published literature, software must be made available to editors and reviewers. We strongly encourage code deposition in a community repository (e.g. GitHub). See the Nature Portfolio guidelines for submitting code & software for further information.

Data

Policy information about availability of data

All manuscripts must include a data availability statement. This statement should provide the following information, where applicable:

- Accession codes, unique identifiers, or web links for publicly available datasets
- A description of any restrictions on data availability
- For clinical datasets or third party data, please ensure that the statement adheres to our policy

The China Kadoorie Biobank (CKB) is a global resource for the investigation of lifestyle, environmental, blood biochemical and genetic factors as determinants of common diseases. The CKB study group is committed to making the cohort data available to the scientific community in China, the UK and worldwide to advance

knowledge about the causes, prevention and treatment of disease. For detailed information on what data is currently available to open access users, how to apply for it, and the timeline for data access (12-16 weeks), please visit CKB website on: https://www.ckbiobank.org/data-access .
Researchers who are interested in obtaining the raw data from the China Kadoorie Biobank study that underlines this paper should contact ckbaccess@ndph.ox.ac.uk. A research proposal will be requested to ensure that any analysis is performed by bona fide researchers and - where data is not currently available to open access researchers - is restricted to the topic covered in this paper.
Further information is available from the corresponding authors upon request.

Human research participants

Policy information about studies involving human research participants and Sex and Gender in Research.

Reporting on sex and gender	The CKB collects information on the biological sex of participants, which was determined based on self-report. All analyses were performed and reported separately by sex.
Population characteristics	Among the 512,724 CKB participants included in this study, the mean age at baseline was 52 (SD 10.7) years, 41% were men and 56% lived in rural areas. Further details of the population characteristics of the study population are reported in the manuscript and in Chen et al., PMID: 22158673.
Recruitment	CKB recruitment is described in Chen et al., PMID: 22158673.
Ethics oversight	Ethical approval was obtained from the Ethical Review Committee of the Chinese Centre for Disease Control and Prevention (Beijing, China, 005/2004) and the Oxford Tropical Research Ethics Committee, University of Oxford (UK, 025-04), and all participants provided written informed consent.

Note that full information on the approval of the study protocol must also be provided in the manuscript.

Field-specific reporting

Please select the one below that is the best fit for your research. If you are not sure, read the appropriate sections before making your selection.

☒ Life sciences ☐ Behavioural & social sciences ☐ Ecological, evolutionary & environmental sciences

For a reference copy of the document with all sections, see nature.com/documents/nr-reporting-summary-flat.pdf

Life sciences study design

All studies must disclose on these points even when the disclosure is negative.

Sample size	The maximum samples available in the CKB were used in this study. All CKB participants (n=512,724) and the genotyped subset (151,347 randomly selected, 16,703 selected as part of nested case-control studies of CVD and COPD which were only included in analyses of relevant outcomes) were included in conventional and genetic analyses, respectively.
Data exclusions	Conventional observational analyses: No exclusion was made for the main conventional analyses. We excluded participants with poor self-reported health or prior chronic disease in sensitivity analyses.<br>Genetic analyses: Genotyped participants with missing data for genomic principal components were excluded. For area-stratified genetic analysis, participants defined as population outliers for the study area based on genomic data analysis were further excluded.
Replication	We did not have a comparable independent dataset available to replicate the findings. However, the observed associations of self-reported alcohol intake with disease outcomes were evaluated with genetic analyses (Mendelian randomization) to assess causal relevance.
Randomization	Randomization was not directly relevant to this observational study design. Covariates were adjusted for in statistical models, and genetic analyses (Mendelian randomization) were performed to minimize potential residual confounding and reverse causation.
Blinding	Disease and hospitalization events among participants were coded by trained staff blinded to baseline information. The data provided to researchers did not contain any personally identifiable variables i.e. datasets were anonymized with uniquely encrypted participant identifiers.

Reporting for specific materials, systems and methods

We require information from authors about some types of materials, experimental systems and methods used in many studies. Here, indicate whether each material, system or method listed is relevant to your study. If you are not sure if a list item applies to your research, read the appropriate section before selecting a response.

Materials & experimental systems

n/a	Involved in the study
☒	☐ Antibodies
☒	☐ Eukaryotic cell lines
☒	☐ Palaeontology and archaeology
☒	☐ Animals and other organisms
☒	☐ Clinical data
☒	☐ Dual use research of concern

Methods

n/a	Involved in the study
☒	☐ ChIP-seq
☒	☐ Flow cytometry
☒	☐ MRI-based neuroimaging
